# Statistics of cortical representational drift can enable robust readout

**DOI:** 10.1371/journal.pcbi.1014297

**Published:** 2026-06-08

**Authors:** Charles Micou, Timothy O’Leary

**Affiliations:** Department of Engineering, University of Cambridge, Cambridge, United Kingdom; University of Osnabrück: Universitat Osnabruck, GERMANY

## Abstract

Representational drift of fixed stimuli, learned tasks and familiar environments is observed in many brain areas, leading to reconfiguration of population codes over days to weeks. This raises the question of whether downstream brain regions employ mechanisms to track changes in population activity and thus preserve the fidelity of the information they extract. We show that the statistical properties of drift have a significant impact on such mechanisms. Over an extended period, a net change in population tuning due to drift can arise from an accumulation of small changes distributed across the population, or via abrupt jumps that affect smaller subsets of cells at each time point. We demonstrate that an adaptive readout can exploit the heavy-tailed statistics of abrupt jumps to maintain a more stable readout using a simple inference mechanism. Using experimental data, we investigate the extent to which heavy-tailed drift statistics are observed during representational drift in the posterior parietal cortex and visual cortex. We find that experimentally measured drift does not conform to a Gaussian random walk. Instead, we find sudden jumps in neural tuning that would be advantageous for a downstream observer adapting to changes in representation. These observations motivate future study to determine whether adaptive decoding mechanisms exist in the brain and to determine the physiological mechanisms that shape the statistics of representational drift.

## Author summary

Percepts, actions and experiences, such as the appearance of an apple and its flavour, are represented in the activity of populations of neurons in various parts of the brain. For decades, it has been possible to measure this activity and relate it to ongoing experiences in animals, including humans. However, recent findings reveal that the activity patterns representing a notionally ‘fixed’ stimulus can change over days to weeks, raising questions about how the brain maintains an internally consistent representation of the world. We show that the statistical properties of these incremental changes can facilitate a stable readout from a stimulus whose encoding drifts over time. Abrupt jumps in activity patterns are easier to discriminate from more subtle, widespread changes, a distinction that becomes more important as the overall rate of change increases. We find evidence of this relationship in two experimental datasets in different brain areas, hinting at potential mechanisms the brain could use to preserve previously-learned information.

## Introduction

A principal function of the brain is to form a model of reality. The neural circuits that underpin this model do not have direct access to the external world. Instead, neural populations must build up and maintain representations of stimuli, environments and events through statistical regularities in upstream activity. However, these statistics are subject to change, which presents an inference problem: did a change occur due to changes in the external world, or due to some internal change in upstream neural circuitry?

Extensive work has shown that while some component of differences between neural activity from one day to the next can be regarded as mean-reverting fluctuations or noise, a significant component corresponds to accumulating changes over a timescale of days to weeks. Such changes are expected if the conditions of a laboratory task are altered, or if an animal’s physiological state or task performance changes. However, a wealth of studies find evidence of neural representations of familiar environments and tasks changing without any obvious evidence of learning or changes in behaviour, a phenomenon known as representational drift [1]. Representational drift has been observed in a variety of brain regions: in the hippocampus [2–4], the visual cortex [5,6], the posterior parietal cortex [7], the auditory cortex [8], and the olfactory cortex [9].

**Fig 1:**
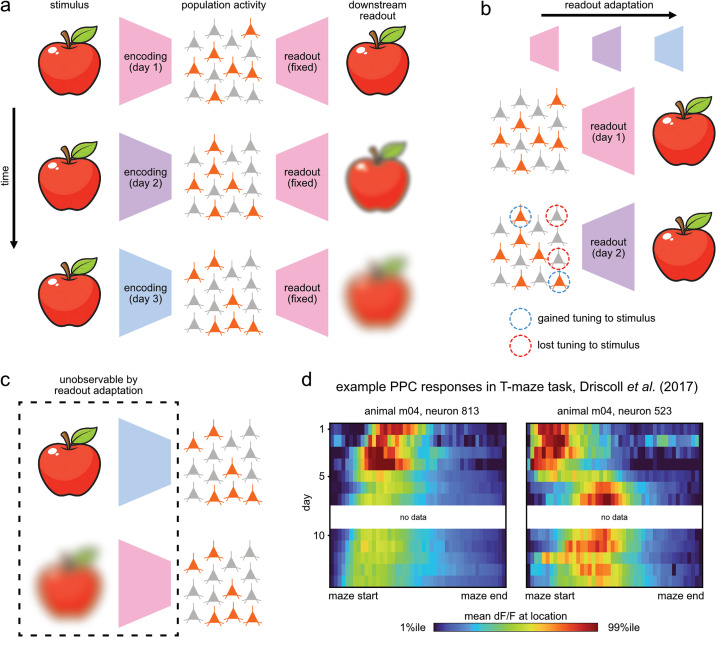
(a) A neural representation describes how patterns of population activity can encode a stimulus. A readout correctly matched to the encoding can infer the stimulus from population activity. However, as the representation drifts, the fixed readout becomes increasingly mismatched, and the quality of the downstream readout gradually deteriorates. (b) A downstream readout can attempt to correct for drift by identifying discrepancies in activity that suggest changes in tuning. This readout adaptation can be performed without supervision by, for example, observing incremental population-level changes in which neurons fire together for the same percept. (c) However, because this adaptation can only observe changes in the neural activity of the population it reads out from, and not factors further upstream, there are instances in which necessarily noisy observations of the outside world can be difficult to distinguish from a drift-induced mismatch between encoding and readout. (d) Ratemaps showing the tuning over the course of 14 days of two example neurons to the position along the main branch of a T-Maze task, as recorded from PPC by Driscoll et al. (2017). Neuron 813 gradually shifts its preferred firing location from one day to the next, while neuron 523 makes a sudden jump from one stable preferred firing location to another.

A simple hypothesis to account for representational drift is to assume that it results from independent, accumulated perturbations to the mapping between neural activity and a specific behavioural measurement. Under this assumption, a fixed readout of population activity would eventually predict behaviour no better than chance (Fig 1a) [10]. This scenario can be interpreted technically, where it has implications for external decoders, brain-machine interfaces and statistical analysis of neural recordings. It can also be viewed as a potential constraint that the brain’s internal circuitry must contend with. This is the scenario we consider here: a neural circuit that receives input from a drifting population must continually adapt its readout to maintain consistent exchange of information [11–13]. Whether and how the brain implements such adaptation remains an intriguing puzzle. Existing work has proposed separately that feedback mechanisms can externally drive correction to readouts [10], that simple Hebbian learning is sufficient to adapt to drift [14,15] and can be supplemented by spontaneous reactivation to strengthen representations [16], and that homeostatic plasticity either in isolation [17] or combined with Hebbian learning [18] can produce stable readouts without the need for an explicit error-correction signal.

These readout adaptation strategies rely on statistical regularities to maintain accuracy in spite of drift: neurons joining or leaving a representation can be identified by changes in the correlation structure of population activity (Fig 1b). However, because downstream readouts can only observe neural activity, and not its external cause (Fig 1c), they must resolve a fundamental ambiguity: is a change in neural activity due to a change in representation, or due to a fluctuation in an uncertain, external world?

While drift is an incremental process at the level of populations, our central hypothesis in this work is that statistical properties of the changes in tuning that take place at the level of individual neurons play a crucial role, both in determining both the maximum drift rates that can be compensated for, and in the amount of noise in observations an adaptation strategy can tolerate. In vivo, and as parameterised by experimental task variables such as location in a maze, we can point to examples of both neurons that change their tuning by gradually altering their preferred firing location from one day to the next, and neurons that instead make sudden jumps between stable preferred firing locations (Fig 1d). We intuit that this latter category, individual neurons with ‘heavy-tailed’ drift statistics that make less frequent but more dramatic changes in tuning, are better-disposed to signal when retuning corresponds to changes in representation, and we therefore predict that they should occur more frequently in drifting populations.

To evaluate this hypothesis, we first present a normative model that is capable of accurately reading out from a drifting population long after a fixed readout would have degraded to chance. This model is an unsupervised ‘adaptive decoder’ that infers changes in how stimuli are encoded in a population. We emphasise that our formulation is not intended to be a faithful description of an algorithm implemented by the physiology of the brain, but instead as a best-case benchmark of such a mechanism. We use this adaptive decoder, which is constrained to rely only on statistical regularities in activity to update its parameters, to illustrate fundamental limitations in the extraction of underlying representational changes from noisy observations, and how these limitations are mitigated by heavy-tailed statistics. We then demonstrate that heavy-tailed statistics present a design trade-off: compensating for drift without these heavy tails requires either larger, more redundant neural populations, or the rates of drift must be slower.

Finally, by considering two existing and previously studied datasets that exhibit representational drift, we explore the potential for biology to make use of these advantageous statistics by exploring whether they manifest in vivo. The first dataset, originally published in Driscoll *et al.* (2017) [7], contains recordings from the posterior parietal cortex (PPC) in mice. PPC is implicated in generating visually guided movements, which can be thought of as representing intended trajectories in navigation and reaching tasks in which animals need to track visual targets or landmarks [19]. Driscoll *et al.* measured neural responses of expertly trained mice during a virtual reality T-maze task. Neural responses formed peaks at specific locations, and drift is visible in single unit tuning on a timescale of days. The second dataset, originally published in Marks & Goard (2021) [6], contains recordings from the V1 region of the visual cortex in mice during repeated exposure to both orientated grating stimuli and movies of naturalistic scenes. Instead of measuring drift through explicitly parameterised tuning curves, this work analysed clusters of neurons with comparable activity and used changes in membership of those clusters to estimate drift rates. Drift in the visual cortex dataset manifests more slowly than in PPC, and is measured in sessions spaced a week apart. We characterise the distributions of tuning changes in both of these datasets and find evidence of heavy-tailed statistics—that drift contains jumps in tuning—especially under high rates of drift.

## Results

### A normative, adaptive decoder operating on an idealised model of drift

We begin by defining drift and describing a normative procedure for decoding from a drifting neural population. We define drift as cumulative changes in population tuning that cannot be entirely accounted for by learning or behavioural observations. In this definition, drift is not simply the result of a stationary noise component in neural signals that can be averaged out. Furthermore, drifting representations do not represent the same variables more precisely or efficiently over time, (e.g., by using fewer neurons). This definition is consistent with empirical characterisations of drift and it implies that a fixed decoder, tuned to decode behaviour from neural activity, will degrade over time.

The objective of an adaptive decoder is to produce an accurate readout from the population that compensates for drift, improving its long term fidelity. Each day, an adaptive decoder updates its estimates of how individual neurons are tuned to stimuli. Outside of initialisation, the adaptive decoder cannot ‘cheat’ with knowledge of stimulus values: it must instead rely purely on statistics of population activity to infer changes in the tuning of individual neurons.

Concretely, we model a neural population of *N* neurons by parameterising their responses $𝐱\in {\mathbb{R}}^{N}$ to a given stimulus $𝐬\in {\mathbb{R}}^{K}$ by defining *P* tuning curve parameters per neuron. The complete tuning of the population is therefore described by $\theta \in {\mathbb{R}}^{N\times P}$. Individual neurons, subject to independent random noise ξ, have their responses defined by an activation function *a* common to all neurons in the population that relates tuning parameters to activity:


$$\begin{array}{c} x_{i}=a(𝐬,\theta_{i})+\xi_{i} \end{array}$$ (1)

**Fig 2:**
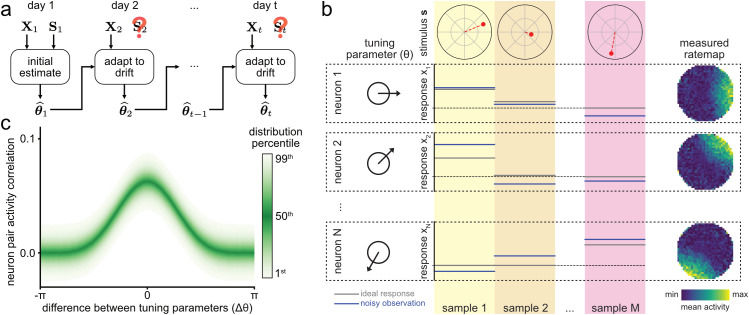
(a) Knowledge of the stimulus ground-truth from an initial experimental session is used to initialise an estimate of the tuning parameters that describe the relationship between stimulus and population activity. Subsequently, and with no further reference to the stimulus ground-truth, an adaptive decoding scheme attempts to keep these estimates accurate while the underlying tuning parameters drift. (b) A model of a neural population that encodes the orientation and magnitude of a stimulus. Each neuron in the population has a tuning parameter that describes its preferred orientation. The population is sampled multiple times during a session, with the activity of each neuron independently affected by noise at each sample. (c) Visualisation of the probability distribution for the value of the correlation between a pair of neurons as a function of the difference between their tuning parameters (simulation parameters M = 10^4^ and σ = 1.0).

In what follows, we will simplify our modelling by treating time as a discrete quantity, where each increment corresponds to a period between an assay of population activity. This brings the modelling in line with the data we will analyse, which is organised into experimental sessions that are performed at least one day apart. On the first day, *t* = 1, we estimate of the tuning parameters ${\hat{\theta }}_{t=1}$ of the population by taking *M* samples of neural activity and the stimulus (${𝐗}_{t=1}\in {\mathbb{R}}^{N\times M}$ and ${𝐒}_{t=1}\in {\mathbb{R}}^{K\times M}$ respectively). On subsequent days *t* = 2,3,...,*T*, the tuning parameters θ change due to drift. The ground-truth values of the stimulus are withheld from the decoder, so our objec*t*ive is to find an adaptive rule that maintains an accurate estimate of ${\hat{\theta }}_{t}$, subject to this constraint (Fig 2a).

For a set of tuning parameters θ, we use the activation function *a*, a prior distribution for stimulus exposure, and a model of the noise process for ξ to construct the function *g*, an approximation of the likelihood of *M* observations of neural activity **X**.


$$\begin{array}{c} p(𝐗|\theta )\approx g(𝐗,\theta ) \end{array}$$ (2)

Naïvely, we could attempt to recover ${\hat{\theta }}_{t}$ from **X**_*t*_ directly by finding the parameters that maximise g(𝐗,θ). However, because the activation function *a* is common to all neurons, the identities of the neurons can be shuffled. The set of parameters maximising g(𝐗,θ) therefore has symmetries that result in degenerate (non-unique) solutions. By considering the tuning of neurons on the previous day, $\theta_{t-1}$, we are able to break these symmetries. In other words, the tuning history of each neuron gives it an identity, providing a well-defined solution to the likelihood maximisation.

We use a stochastic model of how drift perturbs the population from one day to the next, $p(\theta_{t}|\theta_{t-1})$. This produces a maximum a posteriori update rule for tuning in the population.


$$\begin{array}{c} {\hat{\theta }}_{t}=\underset{\theta }{\text{arg}\max}\;ℒ(\theta ,{\hat{\theta }}_{t-1},{𝐗}_{t}) \end{array}$$ (3)

$$\begin{array}{c} \text{where}\;ℒ(\theta ,{\hat{\theta }}_{t-1},{𝐗}_{t})=\log\left(p({𝐗}_{t}|\theta )p(\theta |{\hat{\theta }}_{t-1})\right) \end{array}$$ (4)

Treating drift as a stochastic process that is independent of neuron identity and in which each neuron drifts independently from other neurons, we can express $p(\theta_{t}|\theta_{t-1})$ in terms of a per-neuron drift prior *f*.


$$\begin{array}{c} p(\theta_{t}|\theta_{t-1})=\prod_{i}^{N}f(\theta_{i,t},\theta_{i,t-1}) \end{array}$$ (5)

We then combine our model for observed activity *g* with our description for drift *f* and express the incremental (daily) update rule for $\hat{\theta }$ as:


$$\begin{array}{c} {\hat{\theta }}_{t}=\underset{\theta }{\text{arg}\max}\;\left(\log g({𝐗}_{t},\theta )+\sum_{i}^{N}\log f(\theta_{i},{\hat{\theta }}_{i,t-1})\right) \end{array}$$ (6)

### Statistical regularities in the activity of a simulated population

To illustrate this adaptive decoding strategy, we consider a simple model of a neural population that encodes the direction and the magnitude of a stimulus. This is analogous to neural populations that encode head direction coding in the hippocampal formation and to populations that are tuned to stimulus orientation in the visual cortex (Fig 2b). The stimulus corresponds to a point within the unit circle, defined by a magnitude and direction (i.e., *K* = 2), both of which we draw from uniform distributions:


𝐬=(rϕ)~(U(0,1)U(0,2π)) (7)

Population responses are modelled by assigning each unit a single tuning parameter (*P* = 1), denoting their preferred stimulus angle, and adding independent Gaussian noise to their output.


$$\begin{array}{c} x_{i}=a(r,\phi ,\theta_{i})+\xi_{i} \end{array}$$ (8)

$$\begin{array}{c} \text{where}\;\xi_{i}\sim N(0,\sigma^{2})\; \end{array}$$ (9)

$$\begin{array}{c} \begin{array}{c} \text{and}\;a(r,\phi ,\theta_{i})=\left\{ \begin{array}{cc} r\cos^{2}(\theta_{i}-\phi ) & \text{if }0\leq ∠(\theta_{i},\phi )\leq \frac{\pi }{2}\\ 0 & \text{otherwise} \end{array}\right. \end{array} \end{array}$$ (10)

For convenience in dealing with the circular geometry of this stimulus, we have defined $∠(\psi_{1},\psi_{2})$ as the magnitude of the smallest angle between the angles $\psi_{1}$ and $\psi_{2}$:


$$\begin{array}{c} ∠(\psi_{1},\psi_{2})=|((\psi_{1}-\psi_{2}+\pi )\mathrm{mod}2\pi )-\pi | \end{array}$$ (11)

In this example, the mean decoding error in heading direction is directly equivalent to the mean error in the estimate of the tuning parameters. To construct an expression for g(𝐗,θ), we consider pairs of neurons and the correlation of their activities within a session. Neurons with similar tuning values are more likely to be highly correlated with each other (Fig 2c). The correlation between neurons of a given angular separation is a probability distribution governed by two underlying random processes: the *M* random samples observed on any given day, and the observation noise ξ perturbing those observations (S1a Fig).

Taking *M* to be sufficiently large (S1b Fig, S1 File) that the expected value of the correlation between two neurons of an angular separation Δθ is well-approximated by:


$$\begin{array}{c} E(\Delta \theta )=\frac{1}{6\pi }\frac{1}{16}\left(3\sin2\Delta \theta +2(\pi -\Delta \theta )(\cos2\Delta \theta +2)\right) \end{array}$$ (12)

The distribution of the residual between the expected value and the measured value is normally distributed (S1c Fig). This allows a simple expression for the likelihood of the correlation between the activities **x**_*i*_ and **x**_*j*_ of a pair of neurons with tuning parameters $\theta_{i}$ and $\theta_{j}$ observed *M* times within a session while subject to observation noise with standard deviation σ:


$$\begin{array}{c} p({𝐱}_{i},{𝐱}_{j}|\theta_{i},\theta_{j})\approx h({𝐱}_{i},{𝐱}_{j},\theta_{i},\theta_{j}) \end{array}$$ (13)

$$\begin{array}{c} =\frac{\sqrt{M}}{\sigma \sqrt{2\pi }}e^{-\frac{M({𝐱}_{i}\cdot {𝐱}_{j}/M-E(∠(\theta_{i},\theta_{j})){)}^{2}}{2\sigma^{2}}} \end{array}$$ (14)

Treating the probabilities of pairwise correlations as independent, which is an approximation of p(𝐗|θ), we can then construct an expression for g(𝐗,θ):


$$\begin{array}{c} g(𝐗,\theta )=\prod_{i}^{N}\prod_{j}^{N}h({𝐱}_{i},{𝐱}_{j},\theta_{i},\theta_{j}) \end{array}$$ (15)

When solving the optimisation for ${\hat{\theta }}_{t}$, this amounts to minimising the sum of squared differences between observations of the correlations between neuron pairs and their expected values ***E*** as parameterised by θ.

### Contrasting statistics of drift at the level of individual neurons

In our illustrative model, the need to break symmetries using a drift prior is obvious: while g(𝐗,θ) helps determine the angular differences between neuron tuning parameters, it provides no information about the absolute orientation of any tuning parameter. Knowledge of how the population was tuned on an earlier day, as well as a drift prior describing how that tuning is likely to change over a given interval, is required to compensate for drift.

**Fig 3:**
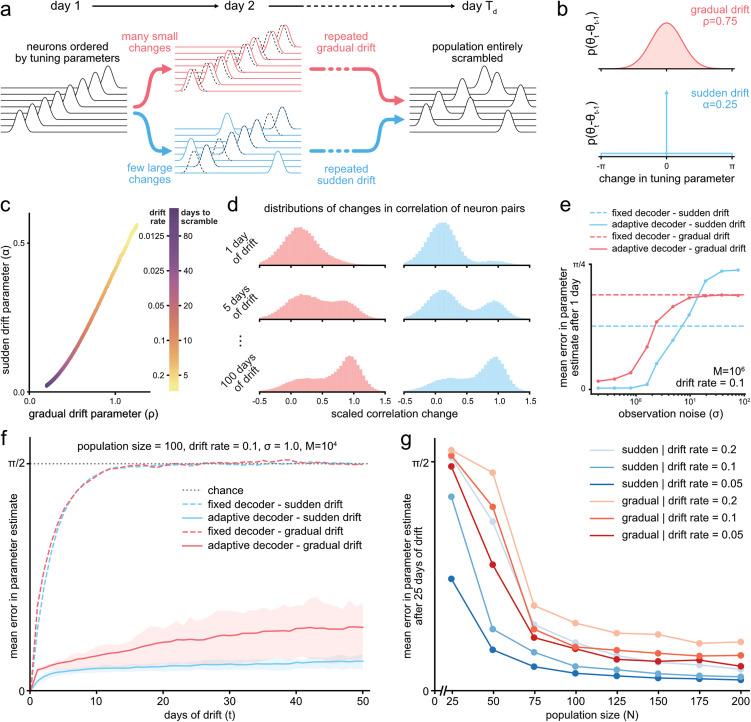
(a) Different underlying stochastic processes at the level of individual neurons, such as either gradually altering all neurons slightly or altering only a few neurons dramatically, can both produce drift. (b) These two example models of drift can be expressed as probability distributions describing the change in tuning parameter from one day to the next. (c) Equivalence between the two drift models determined by the expected number of days required to drive the error of a fixed decoder to within 5% of chance. (d) Histograms for the distribution of scaled changes in correlations between neuron pairs for both the sudden and gradual models of drift, shown for a relatively slow drift rate of 0.02. Only neuron pairs with a correlation of at least 0.05 on the latter day of comparison are displayed. (e) The resulting error in tuning parameter estimates after the first day of drift, shown as a function of the observation noise level σ for both gradual and sudden drift models. (f) Comparison of the accuracy of parameter estimates between fixed and adaptive decoders for both sudden and gradual drift models, both using drift rates of 0.1. Shaded regions show the interquartile range over 100 simulations. (g) The estimation error after 25 days shown for a variety of drift rates (all sufficiently high to scramble the population before 25 days) and for a variety of population sizes. Each datapoint represents the mean of 100 simulations.

Different models of drift can produce similar degradation in the accuracy of a fixed decoder while having very different statistics at the level of individual neurons. We consider two simple yet contrasting models for drift in individual neuron tuning: a ‘gradual’ model, in which all neurons undergo relatively small tuning changes from one day to the next, and a ‘sudden’ (or ‘heavy-tailed’) model, in which most neurons retain their tuning while a small subset retune by making relatively large changes to their tuning (Fig 3a, 3b).

In the gradual drift model, each parameter $\theta_{i}$ moves around the unit circle as if buffeted by Brownian motion. The new value of a tuning parameter measured on a subsequent day is parameterised by ρ, the expected absolute distance drifted. The gradual drift prior, *f*_gradual_, is therefore described by a Von Mises distribution, which is effectively indistinguishable from a Gaussian distribution for the ranges of ρ we use in simulation (S2a-S2b Fig).


$$\begin{array}{c} \theta_{i,t}|\theta_{i,t-1}\sim N(\theta_{i,t-1},\rho^{2}) \end{array}$$ (16)

$$\begin{array}{c} f_{gradual}(\theta_{i,t},\theta_{i,t-1})=\frac{1}{\rho \sqrt{2\pi }}e^{-\frac{(∠(\theta_{i,t},\theta_{i,t-1}){)}^{2}}{2\rho^{2}}} \end{array}$$ (17)

By contrast, in the sudden drift model, each neuron either retains its previous tuning parameter or is assigned an entirely new value. The sudden drift prior, *f*_sudden_, is parameterised by a spontaneous retuning probability α.


$$\begin{array}{c} f_{sudden}(\theta_{i,t},\theta_{i,t-1})=\frac{\alpha }{2\pi }+(1-\alpha )\delta (\theta_{i,t}-\theta_{i,t-1})\;\;\text{ for }0\leq \theta_{i,t}<2\pi \end{array}$$ (18)

Where δ denotes the Dirac delta function.

To allow a fair comparison between sudden and gradual drift models, we calibrate their overall drift rate. We define the drift rate of two models to be equivalent when they reduce the predictive accuracy of a fixed decoder beneath a fixed threshold in the same expected number of days *T*_*d*_ (Fig 3a).


$$\begin{array}{c} \text{drift rate}=\frac{1}{T_{d}} \end{array}$$ (19)

In the case of our illustrative example, we quantify the change in an individual neuron’s tuning between day 1 and day *t* as the angular distance between tuning parameters $∠(\theta_{1},\theta_{t})$. As drift progresses, the expected value of the change in tuning approaches the expected value between the initial tuning parameter $\theta_{1}$ and one chosen entirely at random $\theta_{r}$.


$$\begin{array}{c} \lim_{t\to \infty }E[∠(\theta_{1},\theta_{t})]=E[∠(\theta_{1},\theta_{r})]=\frac{\pi }{2} \end{array}$$ (20)

We describe a population as ‘scrambled’ on some day *T* if it satisfies:


$$\begin{array}{c} \frac{E[∠(\theta_{1},\theta_{r})]-E[∠(\theta_{1},\theta_{T})]}{E[∠(\theta_{1},\theta_{r})]}<\epsilon \end{array}$$ (21)

And define *T*_*d*_ as the first day which satisfies this criterion. This allows us to generate an equivalence between the parameters ρ and α of the two drift models (Fig 3c). We choose ϵ=0.05, noting that the equivalence is not sensitive to this choice of parameter for small ϵ (S2c-S2d Fig).

While the two models of drift provide identical long-term behaviour, with the representation eventually becoming completely scrambled, differences in short-term behaviour have significant impact on the adaptive decoding strategy. The intuition is that a small change in tuning can easily be confused for a noise-driven fluctuation, especially in cases with elevated observation noise (high σ) or limited samples (low *M*), while a large change in tuning is more distinctive. To visualise this, we consider the distribution of changes in the correlation between pairs of neurons. We define the scaled change in correlation across two days *D*_1_ and *D*_2_ as:


$$\begin{array}{c} \Delta C=\frac{C_{D_{2}}-C_{D_{1}}}{C_{D_{2}}}\;\;for\;\;C_{D_{2}}\geq C_{min} \end{array}$$ (22)

In which $C_{D_{1}}$ corresponds to the correlation between the activity of a pair of neurons on *D*_1_ and $C_{D_{2}}$ corresponds to the correlation between the same pair of neurons on the later date *D*_2_. A convenient feature of this metric is that it can easily be computed on experimental data, for which it has two key advantages. First, it does not require parameterising in terms of task variables, which allows us to apply it to different experimental designs. Second, when evaluated over the entire population, it quantifies the same statistical measure—pairwise correlations—that is used by g(𝐗,θ) to adapt the decoder to ongoing drift. We highlight that in constructing distributions of these scaled changes in correlation, we include only pairs of neurons with some minimum correlation *C*_min_ of activities on *D*_2_, as most pairs of neurons are not meaningfully correlated with each other and any changes in their pairwise correlation are therefore driven only by noise. This thresholding introduces a positive bias to the distribution.

Pairs of neurons that whose activities highly correlated with each other on *D*_1_ and remain so on *D*_2_ produce small values of ΔC. Even if both neurons in a pair retain their previous tuning value exactly, the observed value of ΔC is likely to be small rather than zero due to taking a finite number of noisy observations. Such pairs of neurons are visible as a component of the distribution near ΔC=0.0 in simulated models of both sudden and gradual drift (Fig 3d). In the case of sudden drift, we encounter jumps in tuning in which neurons that were not at all correlated with each other on *D*_1_ suddenly become highly correlated on *D*_2_, producing a component of the distribution nearer ΔC=1.0. This results in a distribution that is distinctively bimodal for sudden drift in the short term. In contrast, in the gradual model, the drift in tuning between pairs of neurons takes place more incrementally, with the mass of the distribution gradually shifting away from ΔC=0.0 towards ΔC=1.0 over the course of several days. Eventually, after a sufficient amount of time has elapsed the distributions for both types of drift look identical, corresponding to a distribution of ‘changes’ in tuning between pairs of neurons with completely random parameters. The shape of the distribution at this point is necessarily governed by how neurons tune to stimuli in the population, for example, picking a broader tuning curve increases the odds that a given pair of neurons will correlate with each other. In our simulation this depends on the choice of the activation function $a(𝐬,\theta_{i})$.

### Sudden drift facilitates adaptive decoding

Applying our adaptive decoding strategy to simulated data reveals that the choice of drift statistics at the level of individual neurons impacts the difficulty of discriminating genuine tuning changes from fluctuations due to noisy population activity. On the very first day of drift, the adaptive decoder can accurately infer changes in the tuning parameters of the population for both types of drift under low observation noise ξ (as parameterised by σ). However, as σ increases, adaptive decoders make larger errors when inferring tuning parameters that are subject to gradual drift (Fig 3e).

Adaptive decoders remain accurate long after fixed decoders perform no better than chance. However, because the strategy for estimating parameters relies on using estimates from earlier days, errors can compound and eventually degrade adaptive decoders (Fig 3f).

As the number of neurons in the population increases, adaptive decoders maintain their accuracy for longer for two reasons (Fig 3g). First, while additional neurons requires estimating an increasing number of parameters, the introduction of those neurons provide more neuron pairs with which to narrow the distribution of p(𝐗|θ). The number of additional parameters to estimate grows with *N*, but the number of pairs available grows with *N*^2^, which reduces the likelihood of poorly estimating the tuning of neurons. Second, additional neurons provide redundancy that is relevant over the course of many days of drift: it is statistically inevitable that, eventually, the tuning parameter of an individual neuron will be poorly estimated. With greater redundancy in the population, it is easier to notice an individual poor tuning parameter estimate on some later day, allowing subsequent correction of the estimate and forestalling compounding errors. Sudden drift has an advantage not only because it introduces smaller errors under noisy observations from one day to the next (Fig 3e), but also because it can more effectively use of redundancy to fix error compounding (note the flattening gradient in Fig 3f and the better scaling with population size in Fig 3g), as from the perspective of the adaptive decoder a neuron with a low likelihood of its estimated tuning parameter is no different from a neuron that has spontaneously retuned.

Unsurprisingly, both gradual and sudden drift become easier to correct for under lower rates of drift, as the distribution of changes in tuning is narrower. Nevertheless, for the same population size and the same rate of drift, sudden drift is always easier for an adaptive decoder to compensate for (Fig 3g).

To guard against possible bias when comparing sudden and gradual drift, we checked whether our definition of equivalence of between drift rates impacted our conclusions. We redefined the equivalence between sudden and gradual drift to cause identical degradation in a fixed decoder after the first day of drift. Even though this alternative definition equates situations where sudden drift scrambles populations more quickly than gradual drift, we still find that adaptive decoders maintain accurate readout for longer with sudden drift than with gradual drift (S2e-S2f Fig).

### Is in vivo drift sudden or gradual?

Our computational results imply that neural populations with downstream readouts can facilitate drift compensation by making representational changes stark, especially in the case of fast drift rates. This would allow for populations to adjust faster or employ fewer neurons. Given this difference, we hypothesised that the statistics of in vivo data may bias toward heavy-tailed drift.

To test this hypothesis, we returned to our previous comparison of the distribution of pairwise correlations between neurons (as in Fig 3d). This has the advantage of being agnostic towards which features of environments, stimuli, or tasks a given population encodes, and therefore allows us to draw crude comparisons both between different in vivo datasets and between in vivo datasets and in silico models.

**Fig 4:**
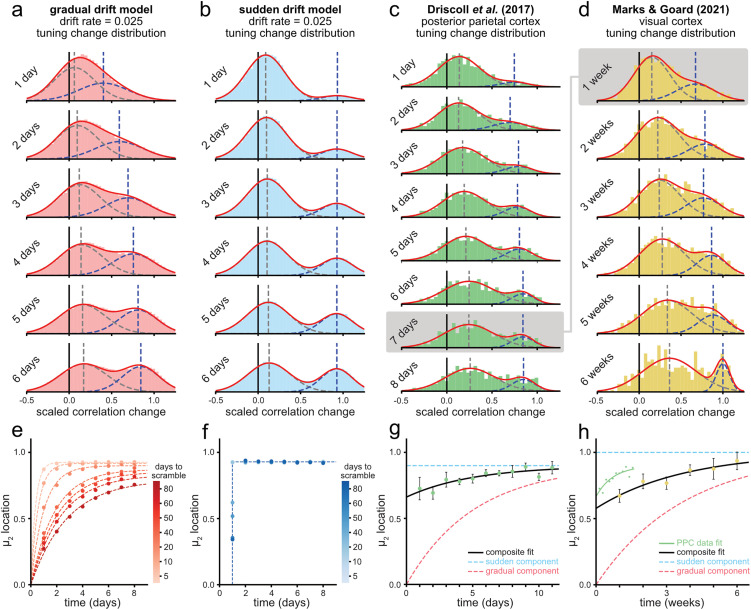
(a) Histograms showing the evolution of the distribution of changes in scaled correlations between neuron pairs in the gradual model of drift over the course of several days. The fit is a 2-component mixture of Gaussians, with each underlying component and its mean shown as dashed lines. (b) As in (a), but shown for the sudden model of drift at an equivalent drift rate and simple final-value fit. (c) As in (a), but shown for in vivo data from the Driscoll et al. (2017) dataset [7]. This comprises recordings from the posterior parietal cortex during performance of a T-Maze task. (d) As in (a), but shown for in vivo data from the Marks & Goard (2021) dataset [6]. This comprises recordings from the visual cortex during exposure to movies of naturalistic scenes. (e) Location of the upper mean (μ 2) in the mixture of Gaussians fit for the gradual drift model shown against time for a range of drift rates. The dashed line indicates a fit to the exponential approach approximation. (f) As in (e), but shown for the sudden model of drift and using a constant value approximation. (g) Location of the upper mean (μ 2) in the mixture of Gaussians fit for the Driscoll et al. dataset. Error bars indicate  ++ /- SEM as estimated via bootstrap. The fit is a weighted combination of gradual and sudden drift models, with each component rendered as a dashed line. (h) As in (g), but shown for the Marks & Goard (2021) dataset.

We reiterate that because both gradual and sudden drift will eventually scramble a population entirely, it becomes impossible to distinguish gradual from sudden drift over timescales that are long relative to the drift rate. In gradual models of drift, the correlation between a pair of neurons gradually increases or decreases as the neurons drift together or apart, while in sudden drift their correlation appears or disappears spontaneously. In the transient period in which it is possible to distinguish gradual from sudden drift, we model the distribution of these pairwise tuning changes as a mixture of two component Gaussian distributions, with one component describing noise-driven fluctuations and the other describing drift-driven changes in tuning (Fig 4a and 4b). While simplistic, this mixture model allows us to qualitatively distinguish gradual from sudden drift. Under gradual drift, the component of this distribution ascribed to changes in tuning gradually separates from the component ascribed to noise. We can therefore identify gradual drift by inspecting the increase over time in the mean of component of the mixture model that corresponds to nonstationary changes ($\mu_{2}$), the behaviour of which can be approximated by an exponential decay towards a final value (Fig 4e). By contrast, under sudden drift, the two components are distinct from the outset, and there is therefore minimal change in the value of $\mu_{2}$ (Fig 4f).

We first considered the dataset of recordings from the PPC in Driscoll *et al.* (2017) [7], where drift is observable on the timescale of days. On visual inspection, changes in the distribution of tuning did not fit exclusively into the category of gradual (Fig 4e) or sudden drift (Fig 4f). While many changes appeared to be mediated by large jumps in tuning, we observed a small growth in $\mu_{2}$ with time, implying that some component of drift is mediated by more gradual changes. We fitted a simple weighted linear combination of our approximations for the sudden and gradual drift models for the evolution of $\mu_{2}$, which attributed relatively more of the distribution to sudden changes (Fig 4g).

Producing the distribution of correlation changes requires neuron pairs to remain intact between imaging sessions and to have well-correlated activity on at least one of the sessions. The distribution in Fig 4f is an aggregate of all animals in the dataset. Some animals had more imaged neurons and more recording sessions (S3a Fig) and drift rates were not necessarily homogenous across animals. Despite this, the proportion tuning changes contributed by each animal remained largely unchanged as a function of separation between sessions (S3b Fig). Therefore, relative changes in drift statistics across animals cannot account for changes in the shape of the distribution.

Next, we considered the dataset of recordings from V1 in Marks & Goard (2021) [6], where drift was much slower and occurred on a timescale of weeks (Fig 4d). As with the PPC data, the distribution is an aggregation over multiple animals with varying amounts of neuron pairs (S3d-S3e Fig). The visual cortex data also contained a mixture of sudden and gradual drift, but in comparison to the PPC data appeared to contain a greater proportion of gradual drift (Fig 4h).

For the PPC, a composite fit allocated 73.6% ± 7.85% (SEM) of the changes in the population to sudden tuning changes (estimated via bootstrap sampling). This contrasted with a smaller proportion of 57.8% ± 8.35% (SEM) in the fit for the V1 data. In this comparison, the population with the slower drift rate appears to have made a greater proportion of its tuning changes gradually. Using bootstrapping, we estimated that the tail of the distribution of changes accounted for a small overlap (*p* = 0.127) between V1 and PPC (see S4e-S4f Fig for distributions). This observation, though preliminary, thus highlights an intriguing hypothesis for future experimental studies to address.

## Discussion

In this work, we developed and tested a hypothesis that the tuning changes in individual neurons underlying representational drift have statistical properties that impact the feasibility of obtaining a robust readout. Our findings can be interpreted in a practical light, in experimental or technological applications where it may be useful to decode neural representations over time without continual reference to behavioural measurements. However, our main motivation was to understand whether measured drift statistics have particular structures that make drift less problematic for neural circuit function than it may appear.

We showed from first principles that sudden drift increments are easier to discriminate—and thus adapt to—than an accumulation of gradual increments. We demonstrated how an adaptive decoder can exploit this in practice using a normative procedure that compensates for changes in tuning by taking into account drift statistics. This model is idealised, and serves only as a plausible best-case for how a neural circuit could adapt to changes. Existing work has explicitly linked a variety of adaptive readout mechanisms to known biological processes, including Hebbian learning and homeostatic plasticity [12,16–18]. Such biologically-rooted plasticity mechanisms will therefore have performance that is bounded by the model we present here.

Our observations motivated the hypotheses that: (1) we should expect to find evidence of sudden drift in experimental data; and (2) the proportion of sudden drift would be higher in circuits with higher overall drift rates. Analysis of data from visual cortex, which has slow drift, and PPC, which has faster drift, was consistent with these hypotheses. We acknowledge the limitations of using data from only two brain regions and, furthermore, that the experiments that generated these data were performed under different conditions during the execution of different tasks. We therefore view our analyses of these datasets and the conclusions we draw from them as preliminary, offering rough agreement with the premise that sudden changes in tuning may be favoured, and that effect should be stronger for higher drift rates. Nevertheless, this is anecdotally corroborated by recent evidence of drift-like changes in tuning in CA1 hippocampus, in which representations make abrupt jumps in tuning on timescales visible within single recording sessions [20].

Our work prompts deeper questions, especially given that our analysis of experimentally measured drift from two datasets found a mixture of gradual and sudden changes. What underlying processes give rise to sudden or gradual drift? Can biological mechanisms enforce a greater proportion of sudden changes in a dynamic population?

Emerging ideas present drift, or at least some component of drift, as a direct consequence of the repeated application of learning rules implemented by neural populations [21,22]. This viewpoint explains drift as a learning-driven walk exploring a degenerate parameter-space [23,24], and existing models of drift therefore assume gradual changes in connectivity. This is either explicit, when introduced as an additional noise process [18], or implicit, when using gradient descent-based learning rules to drive drift in a simulated population [24,25], which can result in Brownian motion-like drift in the parameter-space of populations [26,27]. A well-posed open question is therefore how gradual drift in low-level parameters, such as synaptic weights, give rise to abrupt, sudden changes in tuning at the single unit level.

At least two known phenomena could account for this. The first is simply the nonlinear nature of neuronal responses: gradual weight changes may shift inputs toward or away from a neuron’s threshold, resulting in abrupt appearance or loss of a response. Another explanation relates to observed shifts in excitability of individual neurons over time [14] which may result in similar abrupt appearance of responses. Shifts in preferred tuning, on the other hand, could result from abrupt recruitment and loss of input upstream. In this work, we modelled such sudden shifts in tuning as jumps to new preferred tunings drawn from a uniform distribution. However, if these shifts instead demanded some minimum distance of jump in preferred tuning (i.e., their statistics were even more heavily-tailed), adaptive downstream readouts would be even less likely to confuse shifts in tuning for noise—and this is the main advantage of sudden over gradual drift.

Further modelling work to propose circuit-level mechanisms for avoiding previous tuning values, as well as further experimental work performing simultaneous measurements of responses in a drifting population and a corresponding downstream readout population, are needed to more fully understand the extent to which drift compensation might constrain population-level plasticity.

The impact of sudden vs gradual changes that we described in this work has potential relevance to artificial learning algorithms. Artificial neural networks can generate drift when trained using gradient descent on a continual feed of data. This drift can cause catastrophic forgetting for samples that have remained unseen for long periods of time. While techniques exist to restrain this drift, either by explicitly incorporating penalty terms in loss functions [28], or by actively identifying and utilising subspaces of parameter-space orthogonal to previously learned items [29], we are intrigued by the possibility of mimicking the brain’s strategy of using sudden jumps in tuning to provide an implicit solution. If the dynamics of gradient descent are altered to explore parameter-space by focusing, rather than distributing, updates to network weights, then new samples disturb previously learned representations with statistics that are easier to error-correct in readout layers.

Our findings prompt questions for future experimental work. At minimum, we believe it will be useful to more fully characterise drift statistics, possibly along the lines we have developed here. The datasets we used provide a snapshot of relatively small populations in comparison to entire neural circuits, and do not provide a full picture across multiple circuits in the same animal. It will therefore be informative to revisit the questions we have addressed with larger, within-animal datasets. Furthermore, drift rates measured in vivo are currently known to vary considerably. The discrepancies between fast drift in highly plastic regions, such as the hippocampus [2,3], and slower drift in regions such as the visual cortex [5,6], in addition to evidence that experience [3,4] and task complexity and environment richness can modulate drift rate [30], suggest a strong link between learning rates and drift rates [1,21,22]. These comparisons are drawn qualitatively, across studies and conditions, making it difficult to test whether drift statistics are directly connected to learning rates or unit-level rates of change in tuning. We hope that our work provides motivation for wider measurement and consideration of the unit-level of statistics of drift across regions, as we believe they are integral to understanding how the brain produces steady behaviour with dynamic hardware.

## Methods

### Adaptive decoding and drift simulations

#### Solving the optimisation for the gradual drift model.

We use gradient ascent to solve for the most likely values of ${\hat{\theta }}_{t}$.


$$\begin{array}{c} ℒ(\theta ,{\hat{\theta }}_{t-1},{𝐗}_{t})=\sum_{i}^{N}\sum_{j}^{N}\log h({𝐱}_{i},{𝐱}_{j},\theta_{i},\theta_{j})+\sum_{i}^{N}\log f(\theta_{i},{\hat{\theta }}_{i,t-1}) \end{array}$$ (23)

The left summation depends only on the neuron behaviour model and the right summation depends only on the drift model. In this simple example, we can find analytical forms of the Jacobian and the Hessian, which makes solving via gradient ascent faster. This makes use of two important properties of *h*. First, it is symmetrical over *i*,*j*:


$$\begin{array}{c} h({𝐱}_{i},{𝐱}_{j},\theta_{i},\theta_{j})\equiv h({𝐱}_{j},{𝐱}_{i},\theta_{j},\theta_{i}) \end{array}$$ (24)

Second, in the special case when *i* = *j*, all choices of $\theta_{i}$ are equivalent.


$$\begin{array}{c} \frac{\partial }{\partial \theta_{i}}\left(h({𝐱}_{i},{𝐱}_{i},\theta_{i},\theta_{i})\right)=0 \end{array}$$ (25)

Combining these properties and taking the derivative of the log-likelihood with respect to a single parameter:


$$\begin{array}{c} \frac{\partial ℒ}{\partial \theta_{i}}=2\sum_{j\neq i}^{N}\left(\frac{\partial }{\partial \theta_{i}}\left(\log h({𝐱}_{i},{𝐱}_{j},\theta_{i},\theta_{j})\right)\right)+\frac{\partial }{\partial \theta_{i}}\left(\log f(\theta_{i},{\hat{\theta }}_{i,t-1})\right) \end{array}$$ (26)

And then, to produce the Hessian:


$$\begin{array}{c} \frac{\partial^{2}ℒ}{\partial \theta_{i}\partial \theta_{j}}=\left\{ \begin{array}{c} \frac{\partial^{2}}{\partial \theta_{i}^{2}}\left(\log f(\theta_{i},{\hat{\theta }}_{i,t-1})\right)\text{ if }i=j\\ \frac{\partial^{2}}{\partial \theta_{i}\partial \theta_{j}}\left(\log h({𝐱}_{i},{𝐱}_{j},\theta_{i},\theta_{j})\right)\text{ otherwise } \end{array}\right. \end{array}$$ (27)

We initialise of the parameters for the gradient ascent is to use their values from the previous day, ${\hat{\theta }}_{t-1}$. A complete expression and its derivation is provided in S2 File.

#### Exact solution to the optimisation for the sudden drift model.

The discontinuity in *f*_sudden_ means that it is ill-conditioned for a gradient ascent-based solution applied to the entire parameter space. Instead, we consider a solution that decomposes the problem into smaller components that can be individually solved by gradient ascent (and, in the subsequent section, a technique for approximating this solution for large populations).

For each neuron, there are two possible cases: either the neuron keeps its tuning parameter, or it retunes. In the first case, trivially ${\hat{\theta }}_{i,t}={\hat{\theta }}_{i,t-1}$. In the second case, the neuron needs a tuning parameter update *and* should be withheld from use in estimating the parameters of other neurons this day, as ${\hat{\theta }}_{i,t-1}$ is no longer accurate. To solve, we perform two steps:

- Infer which neurons have kept their tuning parameter intact
- Use this subset of neurons to adapt the parameters of the remaining neurons

Let the set *A* contain the neurons which have retained their tuning parameters. A neuron *i* is a member of this set if and only if $u_{i}=1$, where:


$$\begin{array}{c} u_{i}(𝐗,{\hat{\theta }}_{t-1})=\left\{ \begin{array}{c} 1\text{ if }P(𝐗|\text{changed})P(\text{changed})<P(𝐗|{\text{changed}}^{\prime })P({\text{changed}}^{\prime })\\ 0\text{ otherwise } \end{array}\right. \end{array}$$ (28)

In the concrete example for the sudden drift model, the above condition can be written as:


$$\begin{array}{c} \int_{0}^{2\pi }g({𝐱}_{i},\psi ,{\hat{\theta }}_{t-1})\frac{\alpha }{2\pi }d\psi <g({𝐱}_{i},{\hat{\theta }}_{i,t-1},{\hat{\theta }}_{t-1})(1-\alpha ) \end{array}$$ (29)

Where $g({𝐱}_{i},\psi ,{\hat{\theta }}_{t-1})$ is the probability of the observations **x**_*i*_ given a tuning angle ψ.

This allows us to express the update rule as:


$$\begin{array}{c} {\hat{\theta }}_{t}=\underset{\theta }{\text{arg}\max}\;ℒ(\theta ,{\hat{\theta }}_{t-1},𝐗)≃[\begin{array}{c} z_{0}(\theta_{0},{\hat{\theta }}_{t-1},𝐗)\\ ...\\ z_{i}(\theta_{i},{\hat{\theta }}_{t-1},𝐗)\\ ...\\ z_{N}(\theta_{N},{\hat{\theta }}_{t-1},𝐗) \end{array}] \end{array}$$ (30)

Where:


$$\begin{array}{c} \begin{array}{c} z_{i}(\theta_{i},{\hat{\theta }}_{t-1},𝐗)=\left\{ \begin{array}{cc} {\hat{\theta }}_{i,t-1} & \text{ if }i\in A\\ \underset{\theta }{\text{arg}\max}\sum_{j\in A}\log h({𝐱}_{i},{𝐱}_{j},\theta_{i},{\hat{\theta }}_{j,t-1}) & \text{ otherwise } \end{array}\right. \end{array} \end{array}$$ (31)

Noting that the use of the approximately equal symbol, as this solution discards the small amount of information available in the pairwise mutual tuning between neurons not in *A*.

#### Approximate solution to the optimisation for the sudden drift model.

In practice, evaluating the condition for *u*_*i*_ becomes computationally intractible for large *M* due to resolution limitations of floating point number representations: it requires the product of very many small numbers. Circumventing this limitation in the typical fashion—a sum of logarithms—requires a closed form of the integral of *g*, which is not necessarily available. We instead choose to make an approximation in determining set membership of *A*.

We evaluate $\log g({𝐱}_{i},{\hat{\theta }}_{t-1})$ for each neuron in the population and are therefore able to rank each neuron in the population by conformity to expectation. For a drift rate parameterised by α and a number of neurons in the population *N*, the number of neurons that drifted is binomially distributed. We can select a probability threshold *q* (by using the CDF of the binomial distribution) that results in us excluding the bottom *Q* neurons from *A* by ranking.

The choice of *q* is a hyperparameter. If *q* is large (e.g., 0.95), many of the neurons excluded from *A* are likely to not require retuning. For small *N* and high drift rates (large α), this can exclude a sizeable portion of the population and reduce the accuracy of tuning parameter estimates. Conversely, if *q* is small (e.g., 0.5), neurons that require re-estimating will be included in *A* and not have their parameters updated. In all simulations shown in this work, we set *q* = 0.9.

This approximation has the potential to disadvantage an adaptive decoder compensating for sudden drift when compared to one compensating for gradual drift. Indeed, we observe that levels of noise high enough to make an adaptive decoder perform no better than a fixed decoder, this approximation leads to performance that is worse than rather than equivalent to a fixed decoder (as visible in Figs 3e and S2e). However, given our choice of neuron population sizes *and* the continued outperformance of the sudden drift adaptive decoders, we take our use of this approximation to be immaterial to our conclusions.

#### Implementation of solvers.

The gradient ascent components of these solutions were implemented using the scipy’s library’s optimize.minimize function (in the case of the multivariate optimisation for the gradual case) and the optimize.minimize_scalar function (in the case of the independent scalar optimisations for the sudden case) [31]. We note the additional following details:

- Solution bounds are set to −2π to 2π, and only remapped to the range −π to π after convergence
- The underlying solver method is based on L-BFGS-B [32]
- We use the analytical expression for the Jacobian as described in the supplementary material
- Exact parameters used for the solver, including increases to search depth and stricter tolerances from default usage of these solvers, are available in the code provided as supplementary material

Simulation code is available at: https://github.com/CharlesMicou/heavy-tailed-drift.

### Tuning parameterisation-free drift characterisation

#### Distributions of correlation changes in vivo.

For in vivo data, ‘correlation’ referes to the Pearson Correlation Coefficient. When visualising the distribution of correlation changes for experiments *T* sessions apart (in days for the simulated and Driscoll *et al.* (2017) dataset, in weeks for the Marks & Goard (2021) dataset), we include all session pairs separated by that interval. For example, if *T* = 2 in the Driscoll *et al.* (2017) dataset, we would include day 1 paired with day 3, day 2 paired with day 4, day 3 paired with day 5, and so on. Correlations between neuron pairs can only be evaluated within an individual animal. We aggregate all neuron pair correlations from all animals to produce the distributions.

#### Choice of pairwise correlation threshold.

Lower choices of *C*_min_ are more likely to detect spurious correlations between a pair of neurons as indicative of co-tuning, while higher choices of *C*_min_ result in fewer available pairs of neurons for analysis and a selection of pairings that are potentially less representative of the whole. The vast majority of neuron pairs exhibit little correlation between activities, as visible in the joint distributions of $C_{D_{1}}$ and $C_{D_{2}}$ (S4a-S4b Fig). By considering how these joint distributions change as *D*_1_ and *D*_2_ are separated by greater lengths of time (S4a-S4b Fig), we reason that regions of the distribution corresponding simultaneously to both lower correlations and stationary statistics (no change of the joint distribution over time) correspond to noise rather than drift (whose inclusion would lead to an overrepresentation of tuning changes that appear sudden rather than gradual), and use this to inform our choice of *C*_min_.

We use $C_{min}=0.5$ for both in vivo datasets, taking this to be a reasonable balance between excluding the noise-driven region of the distribution while including a large number of neuron pairs. While this choice of *C*_min_ is necessarily arbitrary, we highlight that our conclusions are not sensitive to the exact choice of value: we can repeat our analyses for a lowered value (e.g., $C_{min}=0.4$) and find our observations largely similar (S4c-S4d Fig).

#### Mixture of Gaussian fits.

We fit the distribution of correlation changes with a 2-component mixture of Gaussians. This is solved using the EM algorithm as implemented by the scikit-learn library [33]. For the in vivo data, we use bootstrapping (1000 iterations per distribution) to fit many resampled distributions to produce the SEM used for the error-bars in Fig 4g and 4h.

#### Modelling the movement of the upper mean of the mixture of Gaussians fit.

In the case of sudden drift, we approximate fits to distribution of correlation changes as always being bimodal with a fixed upper mean (i.e., it is time independent).


$$\begin{array}{c} \mu_{2,sudden}(t)=c \end{array}$$ (32)

In the case of gradual drift, we approximate the upper mean of the mixture model fit to increase as a function of elapsed time, described by:


$$\begin{array}{c} \mu_{2,gradual}(t)=c\;(1-e^{-bt}) \end{array}$$ (33)

For the in vivo data, we fit a weighted mixture of the sudden and gradual models:


$$\begin{array}{c} \mu_{2,composite}(t)=a\mu_{2,sudden}(t)+(1-a)\mu_{2,gradual}(t) \end{array}$$ (34)

Noting the following constraints:


$$\begin{array}{c} 0\leq a\leq 1 \end{array}$$ (35)

$$\begin{array}{c} b\geq 0 \end{array}$$ (36)

$$\begin{array}{c} 0\leq c\leq 1 \end{array}$$ (37)

The parameter *a* is a weighting of importance comparing the sudden component to the gradual component to the model fit, *b* corresponds to the rate of gradual drift, and *c* is a function of the expected correlation between a pair of neurons with tuning parameters selected entirely at random. The parameters *a*, *b*, and *c* are found by minimising the mean squared error between this model of upper mean locations and the experimentally measured values.

#### Relative importance of sudden and gradual drift components.

We perform bootstrapping by sampling with replacement which neurons are included from each dataset. We evaluate 1000 iterations per dataset and estimate the values of the parameters *a*, *b*, and *c*. We report in the main text the SEM of the parameter *a* as evaluated using this process, in addition to the value of *a* obtained when estimated the parameters on the full dataset (i.e., without resampling). Additionally, we test the hypothesis that *P*(*a*_*PPC*_ > *a*_*V*1_) by constructing a distribution of *a*_*PPC*_ − *a*_*V*1_ from our 1000 bootstrap iterations and evaluating the fraction of instances that this quantity is negative. This is visualised in S4e-S4f Fig.

### Drift in the posterior parietal cortex

#### Data inclusion.

The Driscoll *et al.* (2017) dataset comprises recordings from 5 animals (m01 through m05). Because our analysis relies on pairs of neurons being visible across sessions, the number of individual neurons strongly informs the quality of the data. Our analysis includes only neurons that are identified between sessions at the highest level of confidence as labelled in the dataset. Animals m02 and m05 have a distinctly lower total number of neurons identifiable between sessions (S3a Fig), experience a more rapid decline in the number of neurons identifiable between sessions as the number of days between those sessions increase, and exhibit a drop off over time in the number of identifiable neurons. Consequently, while we include all animals in our analysis, animals m01, m03, and m04 are more represented in the aggregated data. The proportion of which animals are representated in the data does not change substantially over time (S3b Fig).

Data are available at the Dryad repository: Driscoll et al “Data from: Dynamic reorganization of neuronal activity patterns in parietal cortex dataset”, https://doi.org/10.5061/dryad.gqnk98sjq.

#### Data processing.

To calculate the activity correlation between neuron pairs during each session, we first convolved spike times (from the original dataset as extracted by deconvolving Calcium traces) with a Gaussian window (standard deviation equivalent to 0.45 seconds). We then evaluated pairwise Pearson correlations between the timeseries of these filtered traces. We did not exclude neural activity during the interval of darkness between trials.

#### Ratemaps.

We produced ratemaps of activity by dividing the T-maze into 10 cm spatial bins along the axis of its main branch and computing the mean dF/F value within each bin, excluding the inter-trial interval. dF/F filtering was applied on a per-session basis. The two ratemaps in Fig 1d are manually-selected examples that illustrate both a gradually drifting neuron and a suddenly drifting neuron within the same population.

### Drift in the visual cortex

#### Data inclusion.

The Marks & Goard *et al.* (2021) dataset comprises recordings from 12 animals (S3d Fig). Mice 7 and 8 are omitted from our analysis as their imaging schedules deviate from the one used by the other animals. Mouse 2 is omitted from our analysis as, uniquely, its surgery provides two separate imaging windows. Our analysis includes only neurons labelled as having a registration quality of 3 or higher (unambiguously identified across sections). Our analysis makes use of only the experimental protocol which exposes animals to the movies of naturalistic scenes. As with the PPC dataset, the proportion of which animals are representated in the data does not change substantially over time (S3e Fig).

Data are available at the Dryad repository: Marks & Goard et al, “Stimulus-dependent representational drift in primary visual cortex”, https://doi.org/10.25349/D9M606.

#### Data processing.

To calculate the activity correlation between neuron pairs during each session, we convolved the raw Calcium traces with a Gaussian window (standard deviation equivalent to 0.25 seconds). These timeseries traces were subsequently used as the basis for pairwise Pearson correlations.

## Supporting information

S1 Fig(a) Visualisations of the distribution of correlations between neuron pairs separated by an angular distance Δθ for a variety of observation noise levels σ and total numbers of samples *M.*The black trace shows approximation for the expected value of the correlation. **(b)** The mean absolute error between the approximation for the expected value of the pairwise correlation and its actual value as computed by a Monte Carlo simulation, shown for in the absence of any observation noise (σ=0) as a function of angular distance between the two neurons. **(c)** Distribution of the error residual, the difference between the expected value of the pairwise correlation and its actual value, in the presence of observation noise (σ=1.0), presented with a comparison to the normal distribution, simulated at *M* = 15.(TIFF)

S2 Fig(a) A visual comparison for a range of gradual drift parameters ρ of the difference between a Gaussian distribution for the change in tuning parameter and the equivalent Von Mises distribution, which captures angles ‘wrapping around’ the circle.These distributions are essentially indistinguishable for small ρ. **(b)** A quantative comparison of discrepancies the Gaussian and Von Mises distributions as a function of ρ using the Kullback-Leibler divergence over the support [−π,π). **(c)** The approach of tuning parameters to their chance values over the course of several days of drift, mean of 10^6^ simulations. Shown for the gradual drift model (left, red) and the sudden drift model (right, blue). Drift rates in the legend listed for ϵ=0.05. **(d)** The equivalence mapping between the gradual and sudden drift parameters for multiple values of ϵ, highlighting the range of gradual drift rates used in the simulations of this article. **(e)** Error in tuning parameter estimates after the first day of drift as a function of the observation noise level σ, using alternative equivalence between gradual and sudden drift that induces the same amount of error in a fixed decoder after the first day of drift. **(f)** As in Fig 3f, but using the alternative drift equivalence.(TIFF)

S3 Fig (a) The number of regions of interest (ROIs) labelled as neurons in each recording session for each animal of the Driscoll *et al.* PPC dataset. **(b)** The proportional contribution of each animal to the aggregated distribution of scaled changes in pairwise correlation. **(c)** Distributions of changes in pairwise correlation for the individual animals, shown for 1, 2, and 3 sessions apart. **(d)** The number of ROIs labelled as neurons in each recording session for each animal of the Marks & Goard V1 dataset. **(e)** As in (b), shown for the V1 dataset. **(f)** As in (c), shown for the V1 dataset.
(TIFF)

S4 Fig (a) Left column: Joint distributions of the correlations between the activity of the same pair of neurons on some day *D*_1_ and some later day *D*_2_, shown for all pairs of neurons in the PPC dataset. The minimum correlation *C*_min_ for neurons to be considered similarly-tuned on *D*_2_ is shown as a dashed line. Right column: changes in density of the joint distribution as *D*_1_ and *D*_2_ grow further apart, relative to the joint distribution when *D*_1_ and *D*_2_ are a single day apart. Small spurious correlations that dominate the volume of neuron pairs are a feature that does not change over time, and so *C*_min_ should be chosen to avoid this region of the distribution (which might otherwise inflate the number of sudden changes in tuning). **(b)** The same analysis as in (a) for the V1 dataset. **(c)** The analysis in Fig 4c repeated with a lower value of *C*_min_. **(d)** As in (c) for the V1 dataset originally shown in Fig 4d. **(e)** Distributions for the value of the parameter *a*, which governs the relative weighting of the sudden and gradual components of the drift in the mixed model, shown for 1000 bootstrap iterations (sampling with replacement of which neuron ROIs from each dataset are included in the analysis). Shown for both the PPC and the V1 dataset. Vertical lines indicate the values of *a* found for the non-sampled version of the data. **(f)** Visualisation of a hypothesis test using the same bootstrapping process as in (e), evaluating the difference in *a* when compared between the PPC and the V1 datasets. *P*(*a*_*PPC*_ ≤ *a*_*V*1_), the fraction of datapoints to the left of the null-hypothesis line, is 0.127.
(TIFF)

S1 File Derivation of the expected value for the correlation of activities between any given pair of neurons in the simulated model. This is used to produce Equation 12 in the main text.
(PDF)

S2 File Derivation of the Jacobian of the simulated model under gradual drift. This is used to produce Equation 26 in the methods section.
(PDF)
