# Supplementary figures and images for: Statistics of cortical representational drift can enable robust readout
Source: PLoS Comput Biol. 2026 Jun 8;22(6):e1014297. doi: 10.1371/journal.pcbi.1014297 (PMC13278673; doi:10.1371/journal.pcbi.1014297)

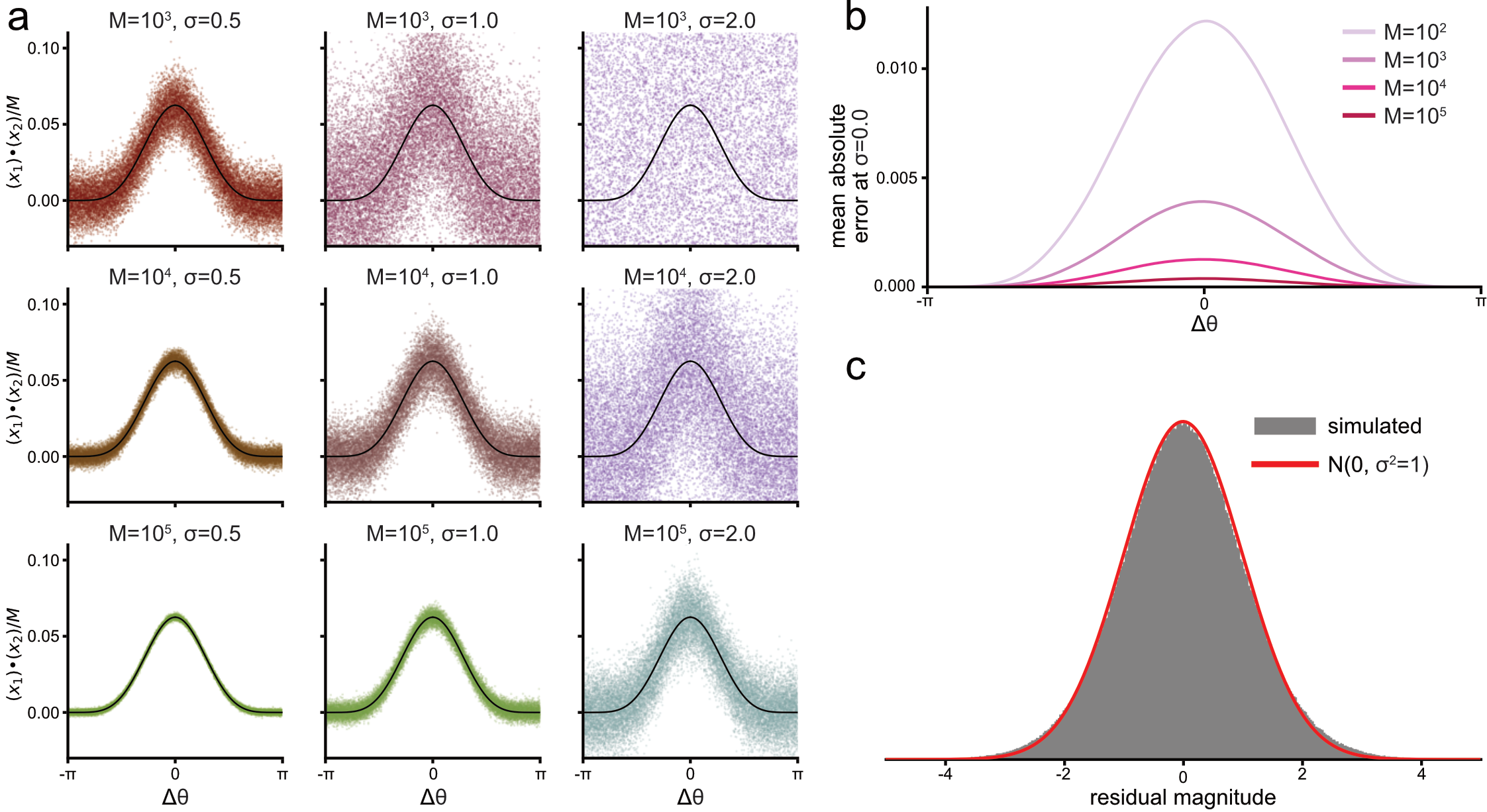

Supplement: S1 Fig — The black trace shows approximation for the expected value of the correlation. (b) The mean absolute error between the approximation for the expected value of the pairwise correlation and its actual value as computed by a Monte Carlo simulation, shown for in the absence of any observation noise (σ=0) as a function of angular distance between the two neurons. (c) Distribution of the error residual, the difference between the expected value of the pairwise correlation and its actual value, in the presence of observation noise (σ=1.0), presented with a comparison to the normal distribution, simulated at M = 15. (TIFF) [file pcbi.1014297.s001.tiff]

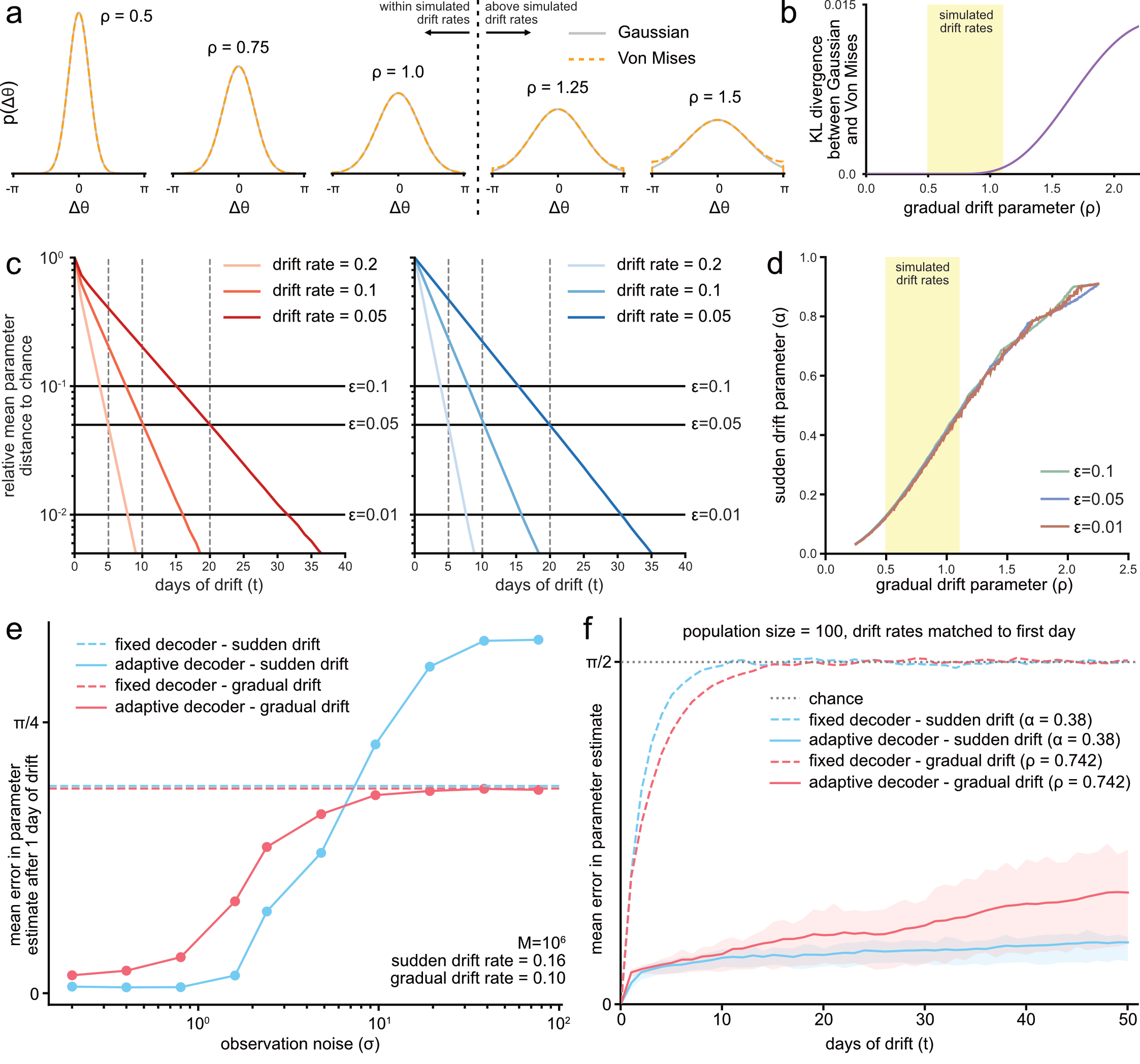

Supplement: S2 Fig — These distributions are essentially indistinguishable for small ρ. (b) A quantative comparison of discrepancies the Gaussian and Von Mises distributions as a function of ρ using the Kullback-Leibler divergence over the support [−π,π). (c) The approach of tuning parameters to their chance values over the course of several days of drift, mean of 106 simulations. Shown for the gradual drift model (left, red) and the sudden drift model (right, blue). Drift rates in the legend listed for ϵ=0.05. (d) The equivalence mapping between the gradual and sudden drift parameters for multiple values of ϵ, highlighting the range of gradual drift rates used in the simulations of this article. (e) Error in tuning parameter estimates after the first day of drift as a function of the observation noise level σ, using alternative equivalence between gradual and sudden drift that induces the same amount of error in a fixed decoder after the first day of drift. (f) As in Fig 3f, but using the alternative drift equivalence. (TIFF) [file pcbi.1014297.s002.tiff]

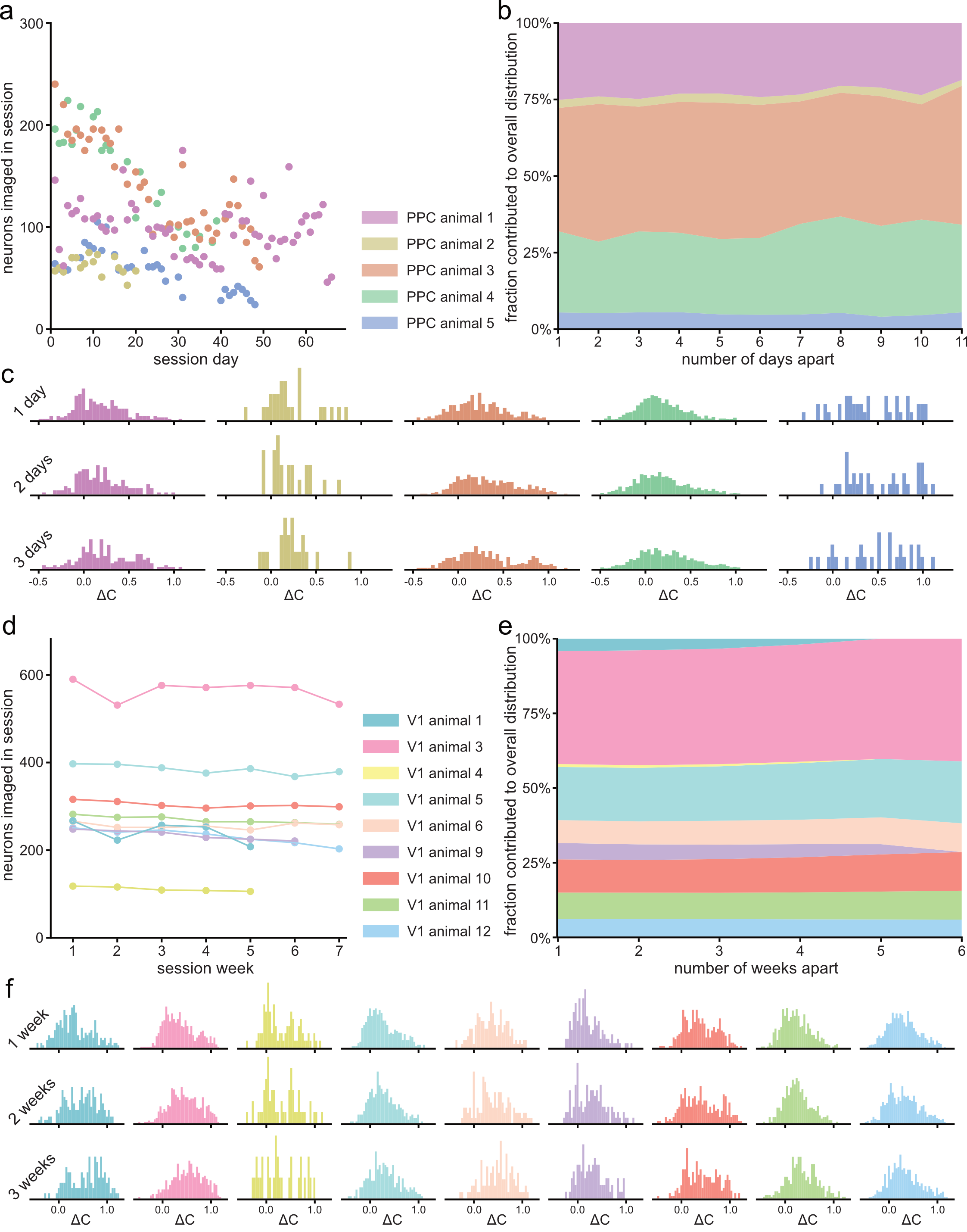

Supplement: S3 Fig — (b) The proportional contribution of each animal to the aggregated distribution of scaled changes in pairwise correlation. (c) Distributions of changes in pairwise correlation for the individual animals, shown for 1, 2, and 3 sessions apart. (d) The number of ROIs labelled as neurons in each recording session for each animal of the Marks & Goard V1 dataset. (e) As in (b), shown for the V1 dataset. (f) As in (c), shown for the V1 dataset. (TIFF) [file pcbi.1014297.s003.tiff]

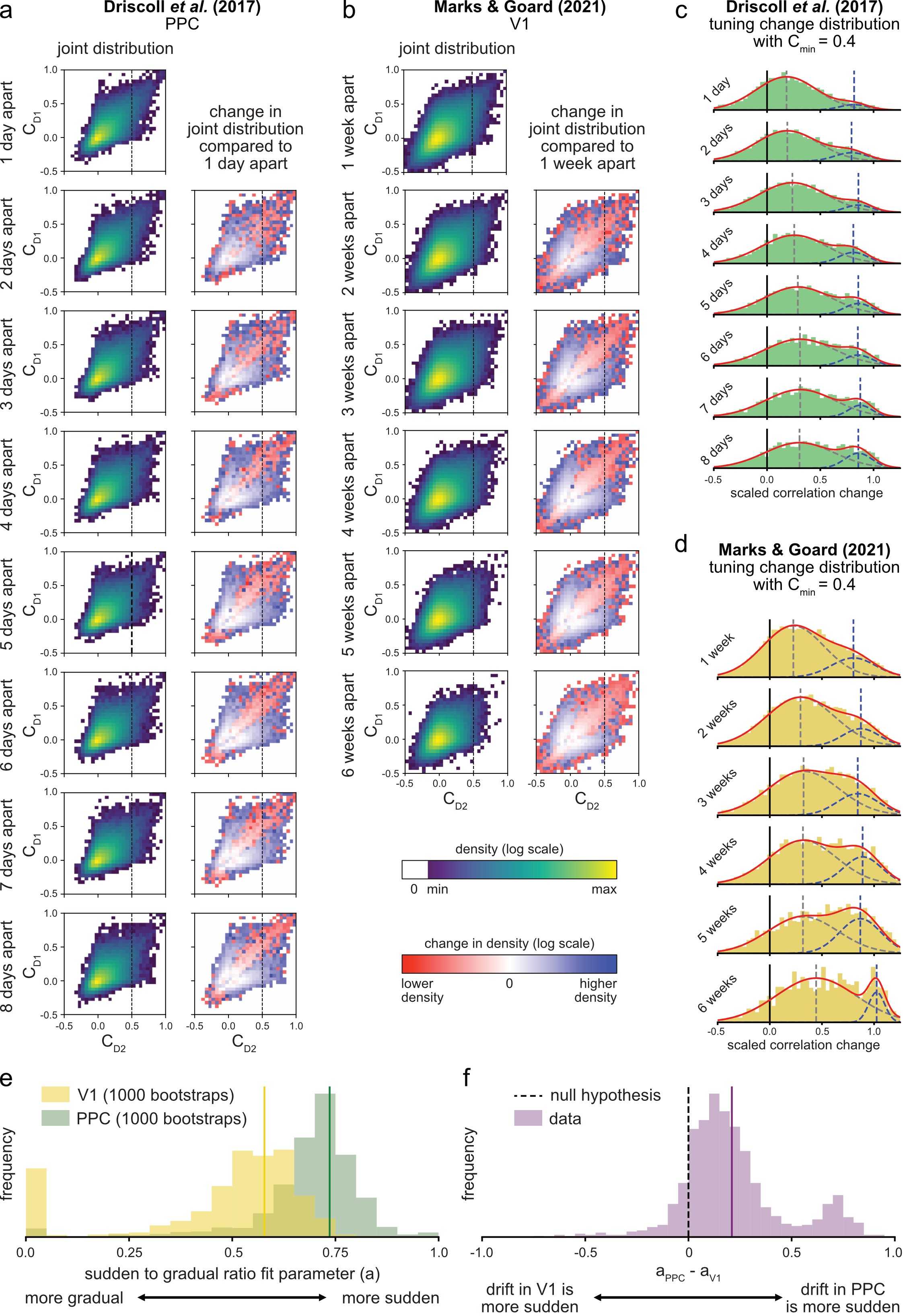

Supplement: S4 Fig — The minimum correlation Cmin for neurons to be considered similarly-tuned on D2 is shown as a dashed line. Right column: changes in density of the joint distribution as D1 and D2 grow further apart, relative to the joint distribution when D1 and D2 are a single day apart. Small spurious correlations that dominate the volume of neuron pairs are a feature that does not change over time, and so Cmin should be chosen to avoid this region of the distribution (which might otherwise inflate the number of sudden changes in tuning). (b) The same analysis as in (a) for the V1 dataset. (c) The analysis in Fig 4c repeated with a lower value of Cmin. (d) As in (c) for the V1 dataset originally shown in Fig 4d. (e) Distributions for the value of the parameter a, which governs the relative weighting of the sudden and gradual components of the drift in the mixed model, shown for 1000 bootstrap iterations (sampling with replacement of which neuron ROIs from each dataset are included in the analysis). Shown for both the PPC and the V1 dataset. Vertical lines indicate the values of a found for the non-sampled version of the data. (f) Visualisation of a hypothesis test using the same bootstrapping process as in (e), evaluating the difference in a when compared between the PPC and the V1 datasets. P(aPPC ≤ aV1), the fraction of datapoints to the left of the null-hypothesis line, is 0.127. (TIFF) [file pcbi.1014297.s004.tiff]
