# Supplementary material for: Statistics of cortical representational drift can enable robust readout
Source: PLoS Comput Biol. 2026 Jun 8;22(6):e1014297. doi: 10.1371/journal.pcbi.1014297 (PMC13278673; doi:10.1371/journal.pcbi.1014297)
Supplement: S1 File — This is used to produce Equation 12 in the main text. (PDF) [file pcbi.1014297.s005.pdf]

## S1 Derivation: Expected correlation between neuron pairs

$$E[\mathbf{x_i x_j}] = \int_{r=0}^{r=1} \int_{\phi=-\pi}^{\phi=\pi} p_r(r) p_\phi(\phi) a(r,\phi,0) a(r,\phi,y) \mathrm{d}\phi \mathrm{d}r \tag{38}$$

We note that $a(r,\phi,\theta)$ is zero for $|\phi-\theta| > \pi/2$. Therefore, taking $0 \le y < \pi$:

$$E[\mathbf{x_i x_j}] = \int_{r=0}^{r=1} \int_{\phi=y-\pi/2}^{\phi=\pi/2} p_r(r) p_\phi(\phi) a(r,\phi,0) a(r,\phi,y) \mathrm{d}\phi \mathrm{d}r \tag{39}$$

$$= \int_{r=0}^{r=1} \int_{\phi=y-\pi/2}^{\phi=\pi/2} p_r(r) p_\phi(\phi) r^2 \cos^2(\phi) \cos^2(\phi-y) \mathrm{d}\phi \mathrm{d}r \tag{40}$$

Noting that $r$ and $\phi$ follow uniform distributions:

$$E[\mathbf{x_i x_j}] = \int_{r=0}^{r=1} \int_{\phi=y-\pi/2}^{\phi=\pi/2} \frac{1}{1}\frac{1}{2\pi} r^2 \cos^2(\phi) \cos^2(\phi-y) \mathrm{d}\phi \mathrm{d}r \tag{41}$$

$$= \frac{1}{6\pi} \int_{\phi=y-\pi/2}^{\phi=\pi/2} \cos^2(\phi) \cos^2(\phi-y) \mathrm{d}\phi \tag{42}$$

$$= \frac{1}{6\pi}\frac{1}{16}\left(3\sin 2y + 2(\pi-y)(\cos 2y + 2)\right) \tag{43}$$
