# Supplementary material for: Statistics of cortical representational drift can enable robust readout
Source: PLoS Comput Biol. 2026 Jun 8;22(6):e1014297. doi: 10.1371/journal.pcbi.1014297 (PMC13278673; doi:10.1371/journal.pcbi.1014297)
Supplement: S2 File — This is used to produce Equation 26 in the methods section. (PDF) [file pcbi.1014297.s006.pdf]

S2 Derivation: Jacobian for gradient ascent in gradual drift model

In order to derive concrete expressions for the analytical form of the Jacobian in Equation 26, we first find the component of $\mathcal{L}$ that depends on the model of neuron tuning:

$$\log g(\mathbf{X}, \boldsymbol{\theta}) = \log \left(\prod_i^N \prod_j^N h(\mathbf{x}_i, \mathbf{x}_j, \theta_i, \theta_j) \right) \tag{44}$$

$$= N^2 \log \frac{\sqrt{M}}{\sigma\sqrt{2\pi}} - \frac{M}{2\sigma^2} \sum_i^N \sum_{j \neq i}^N \left(\frac{\mathbf{x}_i \cdot \mathbf{x}_j}{M} - \mu(|\theta_i - \theta_j|) \right)^2 \tag{45}$$

With the first partial derivative of $\log h$:

$$\frac{\partial}{\partial \theta_i} \left(\log h(\mathbf{x}_i, \mathbf{x}_j, \theta_i, \theta_j) \right) = \frac{M}{\sigma^2} \left(\frac{\mathbf{x}_i \cdot \mathbf{x}_j}{M} - \mu(|\theta_i - \theta_j|) \right) \frac{\partial}{\partial \theta_i} \left(\mu(|\theta_i - \theta_j|) \right) \tag{46}$$

Where:

$$\frac{\partial}{\partial \theta_i} \left(\mu(|\theta_i - \theta_j|) \right) = \frac{1}{24\pi} \frac{\theta_i - \theta_j}{|\theta_j - \theta_i|} \left(\cos(2|\theta_i - \theta_j|) + (|\theta_i - \theta_j| - \pi) \sin(2|\theta_i - \theta_j|) - 1 \right) \tag{47}$$

We then consider the component of $\mathcal{L}$ that depends on the model of drift. For the gradual drift prior, we have:

$$\sum_i^N \log f(\theta_i, \widehat{\theta}_{i,t-1}) = N \log \frac{1}{\rho\sqrt{2\pi}} - \frac{1}{2\rho^2} \sum_i^N (\theta_i - \widehat{\theta}_{i,t-1})^2 \tag{48}$$

With the partial derivative:

$$\frac{\partial}{\partial \theta_i} \left(\log f(\theta_i, \widehat{\theta}_{i,t-1}) \right) = \frac{\widehat{\theta}_{i,t-1} - \theta_i}{\rho^2} \tag{49}$$
