# Supplementary material for: Statistics of cortical representational drift can enable robust readout
Source: PLoS Comput Biol. 2026 Jun 8;22(6):e1014297. doi: 10.1371/journal.pcbi.1014297 (PMC13278673; doi:10.1371/journal.pcbi.1014297)
Supplement: Attachment — Submitted filename: Responses to reviewers.docx [file pcbi.1014297.s008.docx]

**Responses to comments for *Statistics of cortical representational drift can enable robust readout***

**Reviewer #1**

*In this work, the authors begin by discussing representational drift and the idea that adaptive updating of downstream decoders can enable reliable readout despite gradually changing neural codes. They consider two single-cell-level models of drift: gradual drift in the tuning of the entire population, and abrupt, unbounded shifts in random subsets of neurons. Both models give rise to gradual drift at the population level, and their parameterizations allow comparison while controlling for the overall drift rate. The model involving abrupt tuning changes is shown to support significantly better unsupervised decoder adaptation than the gradual model. The authors further analyze two experimental datasets: recordings from primary visual cortex (V1) and posterior parietal cortex (PPC), both collected in studies designed to examine representational drift. By fitting a model combining gradual and abrupt changes to each dataset, they show that the abrupt component is more dominant in PPC than in V1.

Overall, I find this work interesting and timely. The last author has played a key role in conceptualizing and theoretically developing ideas surrounding representational drift and mechanisms for maintaining functionality despite it. The paper is solid, and the integration of theoretical modeling and data analysis to support a coherent hypothesis is much appreciated. While the models are intentionally simplified, as the authors explicitly note, I do not view this as a weakness, since it enables a sharp discussion of the central question. While abrupt single-cell switching in a given brain region has been documented previously, a key and novel contribution of this manuscript is the explicit formulation of representational drift as a weighted combination of gradual and abrupt components, and the use of that mixture model to compare drift regimes across brain areas. This framing goes beyond demonstrating the applicability of unsupervised decoder adaptation, and provides additional lens for asking whether and how different circuits employ fundamentally different modes of representational drift. Several issues require clearer explanation and additional analysis, and some notations would benefit from more precise definition.*

We are grateful for this reviewer’s comments. They covered high-level, conceptual items that we have an opportunity to expand on, identified instances where the provision of additional supplementary material offers greater transparency about choice of methodology, and provided many fine-grained comments on how we could improve the clarity of individual phrases and figure panels. This was a remarkably thoughtful review and we believe the manuscript has been considerably improved by its comments. We address each of the main points below.

*1. The authors focus on analyzing pairwise correlations across neurons and the changes in these correlations between different days. What is the rationale for doing so, rather than examining correlations of the same cells across days (instead of the population of cell-pairs that were above a correlation threshold on the second day of comparison)?
Furthermore, the choice of using scaled correlation while including only cell-pairs whose correlation is above threshold on the later day is not explicitly motivated. This makes the choice appear somewhat arbitrary or even heavily tailored.
Including an illustration of the (2D) distribution of correlations in D1 and D2, and showing how thresholding and scaling yield a bimodal distribution, would help readers appreciate the rationale behind the definition of Delta C used in the paper.*

This is an astute comment and we welcome the opportunity to expand on the choice of this metric. We have considerably expanded the results section where Delta C is introduced to explain our rationale. We reproduce the two main reasons here:

- The adaptive decoder depends on pairwise correlations between neurons to estimate p(x|theta). We wanted an in vivo measure that would be conceptually comparable to our in silico analysis in the first results section of the article.
- Using correlations between pairs of neurons means that we do not have to explicitly parameterise drift in terms of task variables or tuning curves. Given that we compare two in vivo datasets from different brain regions imaged during the performance of different tasks, some parameter-free choice of quantifying drift is necessary. Incidentally, this method is conceptually similar to the original analysis used in Marks & Goard (their methods explicitly make clusters of highly correlated neurons).

The computation of delta C relies on both a threshold and a scaling. We provide a rationale for those separately:

- Thresholding: The correlation matrix for a population of N neurons is NxN, and the vast majority of these entries are approximately zero: the odds of a random pair of neurons being correlated with each other is low. Some neurons may not even be ‘tuned’ to anything during the task. Including all neuron pairs would result in a distribution effectively dominated by noise near zero correlation. We use thresholding to restrict the analysis to neurons that are demonstrably similarly tuned to each other on at least one day, as this makes consideration of changes in tuning tractable
- Scaling: The maximum correlation between any given pair of neurons will vary based on physiology, imaging, and task-specific variables. We are interested in underlying changes in tuning, not an absolute value of correlation. We therefore opted consider proportional changes in correlation, using a scaling strategy that we deemed easy to implement and interpret (scaling by the correlation on the day on which the pair of neurons are guaranteed, by the thresholding step, to have some minimum correlation with each other)

We have also supplied a new supplementary figure (Fig. S4), which shows for both in vivo datasets:

- As requested by the reviewer, the joint distributions of pairwise correlations, shown for the same separations between D1 and D2 as in the main Fig. 4, and marking the location of the Cmin threshold used.
- A visualisation of how this distribution changes as the separation between D1 and D2 increases. We hope that, combined with the joint distributions, this helps illustrate our motivation for Cmin: there is a region of the distribution in which neurons have minimal correlation with each other on both days, and that this region of the distribution does not appear to change over time (i.e. these are spurious correlations driven by a baseline level of noise).
- The Cmin threshold of 0.5, illustrated in these subfigure panels, comfortably excludes that region for both datasets. We deemed it important to choose the same threshold for both datasets.
- We show also, in panels c and d of the same supplementary figure, that our analysis is not sensitive to the exact choice of Cmin, by reducing Cmin by 20% to 0.4 and repeating the analysis from Fig. 4.c and Fig. 4d.

We agree with the reviewer that the choice of Cmin is, at least to a certain extent, arbitrary: Fig. S4 should show that a range of choices of the minimum correlation will exclude neuron pairs that are never co-tuned. However, we hope that in demonstrating that the same analysis holds under a different and more permissive choice of Cmin, we have reassured prospective readers that the choice is not tailored to produce a specific conclusion.

*2. While the bimodality in the distribution of the scaled correlation change is clear in both the simulation and the data, it still needs to be explicitly explained. Readers unfamiliar with this specific kind of analysis may find the reasoning behind the bimodality less clear. It would also be worth mentioning the relationship between bimodality and tuning-curve width (e.g., bimodality would likely be less prominent if tuning were cosine or thresholded cosine, rather than thresholded squared cosine as used here).*

In the same paragraph in which we have expanded upon the choice of Delta C as a metric, we have also attempted to give a more explicit explanation for why the distribution is bimodal.

The reviewer is correct to point out that tuning curve width (relative to rate of drift and to noise) is an important factor in determining the clarity of the bimodal nature of the distribution. In the extreme, a pair of very-sharply tuned neurons with minimal observation noise might appear to drift apart suddenly even if their tuning values had not diverged substantially. We have attempted to clarify this in the text.

*3. The authors have made a much-appreciated effort to include illustrative figures (Fig. 1, 2, 3a–b, etc.). It would be beneficial to have an additional schematic illustrating of the key conceptual point: for two systems in which the same overall level of drift is realized either through sparse abrupt changes or through global gradual changes, online correction by a downstream reader will be easier in the former.*

We have overhauled Fig.1’s illustrative figures in light of this and other comments, and are grateful to the reviewer for pointing these out. We have not included in Fig. 1 an illustration that explicitly proposes that sudden drift will be easier to compensate for than gradual drift, as we find that this is a result that emerges from the result (and becomes more plainly visible in Fig. 3 where it is presented as such).

*4. A notable contribution of this work is its approach to parameterizing aspects of representational drift not captured by standard measures. Specifically, two systems may show the same overall rate of drift, yet one may drift gradually and the other abruptly. This distinction provides a framework for comparing drift across brain in a more nuanced way. It would be valuable to explicitly discuss this conceptual strength. In the analysis, presenting the likelihood of different combinations of gradual and abrupt drift levels (given the data) could illustrate how accurately the contribution of each component can be estimated.*

We agree with the reviewer: the main purpose of this paper is to illustrate that despite the same apparent rate of overall drift, different neural populations may in fact drift differently at the level of individual neurons. We have re-emphasised this in the rewrite of the introduction and the discussion. Furthermore, we have added to the discussion some suggestions of physiologically plausible mechanisms that might explain sudden and gradual changes in tuning in individual neurons.

Our analysis in the original manuscript roughly estimates the relative ratios of sudden to gradual drift in the two in vivo datasets we considered. We have added to Fig. S4 a visualisation, based on bootstrap resampling of the two original datasets, of how accurately we can perform this estimation given the population size and recording duration for each of these populations. We are careful to stress, however, that these datasets vary in a variety of ways—and of particular relevance here is the difference in the task being performed. It may well be the case that different tasks induce different kinds of drift (in addition to different rates), but we do not have the data to speak to this one way or the other, and so while we are able to provide these estimates of different combinations of drift, we would err on the side of caution in extrapolating them beyond the task at hand.

We think that this speaks to the reviewer’s point that, going forward, future experimental observations of drift should seek to characterise not only the overall rate of drift at the level of populations, but also the more fine-grained statistics of individual-neuron retuning.

*5. Using a Gaussian function may not be optimal for circular variables. The authors might consider using a von Mises distribution instead, which would offer a more natural and appropriate representation for this type of data.*

The reviewer is entirely correct. The simulated gradual drift is a von Mises distribution. For the range of drift rates we use in simulation, the Gaussian and von Mises distributions are essentially indistinguishable from each other (the fraction of neurons that drift far enough to wrap around the circle is vanishingly small).

We have clarified in the text that the drift is a von Mises distribution that we approximate as a Gaussian, and we have added two panels to the supplementary Fig. S2 that illustrate (both conceptually and quantified by KL divergence) that these two distributions are in practice interchangeable for the range of drift rates we use in simulation.

Our rationale for using a Gaussian in the analysis, rather than the von Mises distribution, in the analysis is that it lends itself to providing analytical expressions for the Jacobians/Hessians, which considerably improves the speed of our numerical solvers.

*6. The sensitivity of the reader’s adaptation to assumptions about drift parameters and stimulus priors should be further discussed and analyzed.*

The reviewer is correct: the assumptions embedded in the decoder impact its performance. However, we stress that this is a normative model: it shows us how well a neural circuit could perform under ideal conditions and thus represents a reasonable upper bound in performance. Our motivation for focussing on this case was to flesh out a precise hypothesis about drift statistics that we could test in experimental data—other bounds in performance are therefore not relevant to our argument.

In light of this comment, we have revised the language in the manuscript to more heavily stress that this configuration is meant as a normative model.

Minor comments:
 *1. In some places, the definitions should be refined for accuracy. For example:
The text refers to “correlation,” but the equation computes an inner product.*

The reviewer is correct. The original manuscript uses the meaning of correlation common to signal-processing contexts, an inner product, in the modelling section (as above: because is convenient to produce a compact form of the Jacobians/Hessians), but in the in vivo section it also used correlation to refer to the Pearson correlation (a meaning that readers might expect in a statistics context).

We have made a pass through the methods and the main text to make this distinction clearer. We use the signal-processing context as the default, and refer more explicitly to the Pearson correlation for in vivo data.

*T_d is used in eq. 18 to define drift rate, while eq. 20 states “the population is scrambled on day T_d”; for consistency, clarify that T_d is the earliest day on which the population is scrambled.*

We have now clarified the wording around T_d.

*x_i and x_j are sometimes referred to as neurons (page 6), though they are defined as rates.
In eq. 12, it is unclear whether the probability refers to x_i,x_j themselves or to the similarity between them.*

Based on this comment, we have cleaned up the wording surrounding x_i and x_j and attempted to disambiguate any instances in which we refer to their mutual similarity.

*It is unclear whether sigma in eq. 9 and eq. 14 denotes the same parameter.*

These are the same parameter and we have now made this more explicit in the main text.

*Given independent noise across neurons (eq. 1), the notation should likely be xi_i, not just xi.*

This is an excellent suggestion and we have incorporated it in the relevant equations (eqn 1 and eqn 8).

*2. The introduction says “and how these limitations are mitigated by heavy-tailed statistics”, at this early point of the paper, it may be worth explicitly stating that the statement refer to.*

We have considerably reworked the introduction with this (and other) comments in mind, and now introduce the idea of heavy-tailed statistics prior to the sentence this comment references.

*2. Provide specific examples of single cells from the datasets illustrating gradual vs. abrupt drift, even if only for illustrative purposes.*

We thank the reviewer for their input on this. We have reworked Fig. 1 substantially with this in mind, and we now also include in vivo examples of individual neurons undergoing both sudden and gradual changes in tuning over the course of several days (Fig. 1D). We picked cells from the PPC dataset, rather than the V1 dataset, as these can easily be parameterised in terms of a task variable (track position) that allows us to visualise incremental adjustments of the tuning curve.

*3. The manuscript states that “representational drift generically leads to a gradual degradation in the accuracy of a fixed readout… eventually rendering that readout no better than chance.” This is true for some reports but not all; some studies have shown preserved decoding despite drift. (Still, adaptive readout updates are important either way.)*

We agree with the reviewer that this requires more nuance and have clarified in the introduction the eventual degradation to chance depends on some assumptions about the overall statistics of drift.

*4. The phrase “Finally, we explore whether biology makes use of these advantageous statistics…” may suggest a mechanistic claim (that biology uses the statistics), rather than an observational one. Consider replacing “makes use of” with “exhibits” or “manifests.”*

Fixed.

*5. When PPC is first mentioned, briefly describe it or its functions (Unlike “visual cortex,” its role is not self-explanatory).*

We have added a brief description of the PPC for additional context.

*6. Figure 1a is clear, but Figure 1b is not. It seems to conflate two ideas, the reader’s update and the ambiguity between noise and representational drift.*

We have split out Figure 1b in the original manuscript into two panels in the revised manuscript (1b and 1c). This now illustrates the two ideas separately.

*7. The phrase “nonstationary statistics” is technically correct (as distinct from noise) but may be misleading, since drift may still have stationary statistics in some senses (unlike learning).*

We have removed the phrase ‘nonstationary’ from the introduction and discussion, where we make more general comments about drift in the literature. We have kept references to ‘stationary’ and ‘nonstationary’ statistics in a small number of instances in the results and methods sections, where they are a concise and technically accurate description of the work.

*8. In Fig. 1b., the horizontal dashed lines’ meaning is unclear and they resemble the neuron frames too closely.*

We have added a legend to the new version of Fig. 1b, in addition to the other revisions to Fig. 1 suggested above.

*9. For the notation for smallest angle between psi_1 and psi_2, defining it in an equation instead of an inline definition, would be clearer.*

Done.

*10. For eq. 11, include a reference to the supplementary derivations.*

Done.

*11. “knowledge of how the population was tuned on an earlier day, as well as a drift prior describing how that tuning is likely to change, is required to anchor the system to an absolute frame of reference.” I would guess that the assumption of drift being generally slow may be as important as specific priors.*

While the rate of drift is encoded as part of the prior, this comment highlights a detail that we should have made more explicit in our original manuscript. Drift must be sufficiently slow that when measured over a time period such as a recording session, tuning must be sufficiently stable to allow the assessment of how pairs of neurons have changed in tuning relative to each other since the last recording session. We have revised our manuscript, immediately after equation 1, to clarify that we assume for modelling purposes that drift takes place sufficiently slowly that we can consider time as discrete (i.e. that the vast majority of drift takes place between one imaging session and the next, rather than within a single imaging session).

*12. In some figures (e.g., 2a, 3a), day 1 uses the ground truth, while in others (e.g., 3d, 3e) there appear to be errors already on day 1 (so initialization is at day 0). Clarify for consistency.*

We thank the reviewer for pointing this out. The text uses day 1 as ground truth, and there were indeed instances where the figures started counting from day 0. We have opted to make all instances refer to day 1 as the day with the ground truth. Consequently, we have adjusted the labels in the figures, most notably Fig 3e (and its equivalent in Fig. S2), to read “after 1 day of drift”. We have kept the ‘days of drift’ labelling scheme for Fig. 3d and Fig. 3f (and their equivalents in the supplement), so that the plot starts at x=0 (with 0 days of drift ‘applied’), as we found this visually clearer than starting at x=1.

*13. The phrase “the two models of drift provide identical long-term behaviour”: does it mean convergence to the same steady state, or identical functions of time for large t? Clarify the intended meaning.*

We have modified this sentence in the latest version of the manuscript to clarify our intended meaning: that both models of drift will eventually scramble a population to the point where it appears, from the perspective of some initial day, that the tuning parameters have been selected entirely at random.

*14. After eq. 21, explicitly explain the notations C_D1 and C_D2.*

Done.

*15. The sentence “changes in the distribution of tuning do not fit neatly into the mould of either sudden or gradual drift (Fig. 4f)” could be revised to: “changes in the distribution of tuning do not fit neatly into the model of either sudden (Fig. 4e) or gradual drift (Fig. 4f)”.*

We have revised this sentence.

*16. Change some phrases to be clearer: “and to be have well-correlated activity” to “and to have well-correlated activity”; “do not have direct access external world” to “do not have direct access to the external world”; and “To explore whether in-drift manifests drift suddenly or gradually” to ”To explore whether (in-vivo?) drift is manifested suddenly or gradually”.*

We have revised each of these sentences.

*17. The statement “The population with the slower drift rate makes a greater proportion of tuning changes gradually”: clarify what was statistically tested, and consider softening the claim given the limited significance (as it wasn’t significant in one analysis and p values was 0.034 in another).*

We have revisited this statistical analysis and rewritten the methods accordingly. In the original manuscript, we quoted the values for two standard t-tests (one assuming a pooled variance, and the other not) applied to the same data—while either would be appropriate, we chose to provide both, as we wanted to highlight that our value might fall either side of a standard significance threshold, depending on the exact choice of method (favouring transparency over simply quoting the one that happened to “pass” the test).

In the revised version of the manuscript, we use a simple bootstrapping test to determine whether drift is more sudden in the PPC dataset than in the V1 dataset (detailed in full in the revised methods). This test is less powerful and produces a ‘worse’ p value (p=0.127), but its main merit is producing a bootstrap distribution we can visualise (we provide this in Fig. S4e-f). We hope that this visualisation provides even greater transparency to prospective readers and helps illustrate the nature of the bootstrapping method used to provide the comparison.

In accordance with the ‘not significant’ p value, we have softened our language around the strength of the observation that the faster-drifting population drifts more suddenly.

*18. Maybe change the phrase “an argument and computational simulations” to “an analytical argument and simulations” (as any simulation is computational).*

Fixed.

*19. In the phrase “this advantage is more pronounced in situations involving higher drift rates”, would it be correct to mention both higher drift rates and smaller population (as also discussed in Fig. 3g)?*

The reviewer is correct that our predictions from Fig. 3g suggest that adaptive readouts that draw on a larger population of neurons would be able to tolerate higher rates of drift than ones drawing from a slower population. However, when we draw comparisons to in vivo datasets, the populations that are imaged are only small subsets of actual brain regions. Without either imaging data at unprecedented scale or making (perhaps tenuous) arguments based on inferred connectivity between populations, we would only be speculating on how our predictions connect to experimental observations of drift.

We therefore prefer, in the scope of this work, to keep our arguments about the balance of sudden to gradual drift in terms of overall drift rates, as this is something that we can comment on with these datasets. Nevertheless, the reviewer is correct to point out that this is an eventual prediction that might be technically tractable to verify in vivo much further down the line and we have signalled this as an avenue for future work.

*20. The important point that an observation of gradual drift at population-level (and after averaging across-animal and across day-pairs averaging) does not necessarily imply gradual drift at the single-cell level appears only in the final paragraph. It deserves mention earlier in the paper, possibly in the Introduction.*

Motivated by this and the comments in point 4, we have put more emphasis on this point. This includes the explicit distinction between real observed neurons in the new Fig. 1d.

**Reviewer #2**

*Representational drift is a common observation across many brain regions. If neurons continually change their tuning properties, how can downstream circuits correctly interpret their activity? Micou and O’Leary address this question by proposing that the brain can exploit stimulus-evoked correlations between neurons to continually infer shifting tuning properties in an unsupervised manner. Using a simple model, they compare two forms of representational drift: (i) gradual, approximately homogeneous drift across the population, akin to Brownian motion; and (ii) abrupt, large changes restricted to a small subset of neurons. They show that their proposed mechanism performs better under the latter form of drift. Finally, using neural data from two brain regions, they argue that the experimentally observed drift is more consistent with the sparse, abrupt type.

Overall, I found the paper original and interesting. My comments are primarily minor, but addressing them can improve clarity and precision.*

We are very grateful for this reviewer’s comments. They have provided several opportunities to make the modelling component of the article more technically precise, and in one instance pointed out an important technical inaccuracy in how the text characterises one of the distributions (the incorrect description of the gradual drift an Ornstein-Uhlenbeck process), which we are very glad to be able to rectify. We address their comments individually below.

*Page 3:
The sentence beginning “Drift is distinguished from the other form of nonstationary changes…” is very long and difficult to parse. Its meaning is unclear to me. I recommend breaking it into multiple shorter sentences.*

We agree with this comment and have revised both this sentence and the surrounding paragraph for clarity.

*Pages 3–4: Notation
The use of boldface notation for vectors is inconsistent. For example, (\mathbf{s}) and (\mathbf{x}) are vectors and are typeset in bold, but (\theta_i), which appears to be a vector in (\mathbb{R}^P), is not bolded in Equation (1). If (\theta_i) is intended to be a vector, it should be written as (\boldsymbol{\theta}_i) for consistency, unless there is a conceptual reason for the distinction that should be clarified.*

We are grateful to the reviewer for pointing out this inconsistency. We have added boldface to equation 1, where it is correct. We have kept the boldface unapplied to equation 8, as at that point in the work we have defined P=1 for our concrete example, rendering it effectively a scalar at that point.

*Page 6:
The gradual drift is described as an Ornstein–Uhlenbeck (OU) process. However, OU dynamics revert to a mean, whereas Brownian motion on the circle does not. If the intended process lacks mean reversion, then describing it as OU is inaccurate. Please clarify the stochastic dynamics underlying the “gradual” drift condition.*

The reviewer is entirely correct. The description in the original manuscript as an OU process was inaccurate and we have removed any reference to it. We have kept the ‘Brownian buffeting’ language, as this is an accurate description of the statistics and a helpful metaphor.

We have updated the text surrounding this section, and clarified that the ‘true’ distribution (Brownian motion on the circle) is a Von Mises distribution. However, for the rates of drift we consider in this article, the Von Mises and Gaussian distributions are effectively indistinguishable from each other—for clarity we have included an additional pair of panels in the supplement (Fig. S2a and S2b) that both qualitatively and quantitively compare the similarity of the Von Mises/Gaussian distributions for our range of simulated drift values. We use the Gaussian as our drift prior, as it makes an analytical form of the Jacobian/Hessian easy to derive, and having these available considerably improves the performance of our numerical solvers.

*Equations (16) and (17):
The quantities (f_{\mathrm{gradual}}) and (f_{\mathrm{sudden}}) appear in the equations but are not introduced in the text. A brief explanation of what these functions represent would help readers follow the development of the model.*

We have now clarified the notation for f_gradual and f_sudden in the section that precedes this equation.

*Equation (17):
The “0 otherwise” clause seems unnecessary, since the preceding line already specifies the value for all (\theta_{i,t}).*

Fixed.

*Equation (20):
The definition of T_d with inequality is inaccurate. Either use equality, or define T_d as the minimal number of days such that the inequality is satisfied.*

We have more strictly defined T_d in the revised version of the manuscript, using the reviewer’s suggestion as using the minimal number of days to satisfy the inequality.

*Discussion

I found the arguments in the first paragraph of the Discussion section and in the in-silico section of the Discussion sections confusing. There is no evidence that representational drift is a feature and not a bug of the system. The authors have shown that abrupt changes in few neurons are less detrimental than gradual ones in all neurons. However, to argue for the “advantages of …changes in representation”, and their possible role in silco, one first needs to show convincing evidence that these changes are advantageous in the first place.*

We have rewritten the Discussion to make these philosophical points clearer. We agree that we cannot determine whether drift is a bug or a feature, and we hope that the writing more clearly reflects this. We would add that in continual learning, *some changes* need to occur to enable learning, so in this sense the changes are a constraint, not an ‘advantage’ per se.

*Additional Suggestion

An intuitive explanation might help readers before introducing the formalism. Suppose the neural population is large and noise levels are low. At each time step, only a small fraction of neurons undergo substantial tuning changes. Because these changes induce noticeable shifts in their correlations with other neurons, these neurons are easy to identify. Their new tuning curves could then be inferred by examining the tuning of the neurons with which they are now correlated. This intuition may provide a helpful conceptual bridge to the correlation-based inference approach used in the paper.*

We thank the reviewer for these suggestions: this is the exact insight we wish to convey to readers. We have attempted to improve this in two places. First, in the introduction, we have reworked Fig. 1 to separate out two concepts into separate panels (b and c): (1) that changes in tuning might be inferable by monitoring which neurons are and are not correlated with each other, (2) in the presence of noise in observations introduces the risk of mistaking noise for a change in tuning. Second, we have considerably expanded the section in which we define the scaled correlation change metric, which serves as a bridge between the computational and in vivo analyses and should serve to highlight that changes are easier to identify under sudden drift.

As an additional point, the revised version of the manuscript also comments in the discussion that it would be possible to construct even ‘heavier-tailed’ distributions than the sudden drift model we used in simulation: if retuning neurons could, through some hypothetical mechanism, prevent themselves from retuning to their previous tuning value after a retuning event (i.e. ensuring some minimum distance of tuning jump), their tuning changes would be even more distinctive and therefore enable even better adaptive decoding.

**Reviewer #3**

*In the manuscript, “Statistics of cortical representational drift can enable robust readout,” the authors propose an adaptive decoding scheme which allows one to overcome the degradation in readout due to representational drift (RD). The scheme is meant as an ideal normative solution to drifting population codes, and not as a mechanistic description of a physiological mechanism for readout. The main finding is that, given a fixed amount of drift over a certain period of time, the decoder works better if some neurons undergo significant drift and others much less, compared to the scenario where all cells undergo a bit of drift. Analysis of data from parietal and visual cortices reveal a mixed bag, where neither the “all-a-little-bit” Gaussian model, nor the broad-tailed “stay-or-change-a-lot” model capture the statistics of drift alone (as characterized by changes in pairwise correlations). Rather, a mixture of the two does well, with broad-tailed changes in tuning doing better when drift is fast.
What a pleasure it is to read a well-written paper, with clearly expressed ideas and an interesting and not-overstated main finding. This is an important contribution to the literature on RD and I have relatively little to say beyond that.*

---

*I do, however, have some thoughts on the relationship between the mechanisms responsible for drift, and the changes in the parameters “theta” in this manuscript. This is something the authors very clearly acknowledge is -not- the topic of the current manuscript, however I believe it is germane to the conclusions drawn. Namely, at least for the data sets analyzed, the authors can rule out simple Gaussian statistics for the RD, particularly for the parietal cortex. However, the observed changes in cells’ tuning are likely due to either synaptic plasticity or single-cell excitability, both of which will affect the cells’ tuning in nonlinear ways. Put another way, assuming changes in a cell’s input follow Gaussian statistics, it is quite possible that the consequent changes in tuning are -not- Gaussian. At least in the case of a cell’s firing rate, it is known that a Gaussian distribution of inputs actually results in a log-normal, i.e. long-tailed, distribution of firing rates. Recent work with spiking networks (Devalle et al, Sci Rep 2025) showed that Gaussian changes in inputs are sufficient to provide a quantitative fit to data from hippocampus, though perhaps importantly pairwise statistics were not studied. So there is some chance that simple Gaussian statistics at the mechanistic level of description, combined with whatever nonlinearities transform this into changes in the “thetas” could account for RD in the data sets looked at here.
That’s it. I’m very happy to hear the authors’ thoughts on this, whether or not they feel it warrants any change to the main text.*

We are grateful for this reviewer’s generous comments on the virtues of the manuscript, and are glad to entertain some further discussion on the underlying physiological mechanisms that might give rise to sudden vs gradual drift. We have added some points to this effect in the discussion section of the revised manuscript, which we believe to quite closely follow the reviewer’s line of reasoning

To respond directly here: yes, we agree completely, gradual (Gaussian) weight changes can generate apparent jumps in tuning. Neurons are by nature non-linear components, and so abrupt changes to output can arise from gradual changes to input (e.g. gradual shifts in synaptic weight, or in afferent activity). Something like this could explain the sudden dropping in and dropping out observed in certain experimental datasets, but so too could shifts in excitability of individual neurons over time. Changes in preferred tuning via abrupt jumps, rather than simply dropping in or dropping out, may also result from gradual changes in the synaptic weights due to the reasoning above, or could result from shifts in recruitment of upstream neurons.

*Minor typo: Fourth line from the end on page two, “used changed in” should be “used changes in” I believe.*

Fixed, thank you.
