# Supplementary material for: Statistics of cortical representational drift can enable robust readout
Source: PLoS Comput Biol. 2026 Jun 8;22(6):e1014297. doi: 10.1371/journal.pcbi.1014297 (PMC13278673; doi:10.1371/journal.pcbi.1014297)
Supplement: Peer review history

# Peer review: Decision Letter 0

**DOI:** 10.1371/journal.pcbi.1014297.r001

**Authors:** Tim Kietzmann

31 Dec 2025

-->PCOMPBIOL-D-25-01957

Statistics of cortical representational drift can enable robust readout

PLOS Computational Biology

Dear Dr. O'Leary,

Thank you for submitting your manuscript to PLOS Computational Biology. After careful consideration, we feel that it has merit but does not fully meet PLOS Computational Biology's publication criteria as it currently stands. Therefore, we invite you to submit a revised version of the manuscript that addresses the points raised during the review process.

Please submit your revised manuscript by Mar 02 2026 11:59PM. If you will need more time than this to complete your revisions, please reply to this message or contact the journal office at ploscompbiol@plos.org. When you're ready to submit your revision, log on to https://www.editorialmanager.com/pcompbiol/ and select the 'Submissions Needing Revision' folder to locate your manuscript file.

Please include the following items when submitting your revised manuscript:

* A letter that responds to each point raised by the editor and reviewer(s). You should upload this letter as a separate file labeled 'Response to Reviewers'. This file does not need to include responses to formatting updates and technical items listed in the 'Journal Requirements' section below.

* A marked-up copy of your manuscript that highlights changes made to the original version. You should upload this as a separate file labeled 'Revised Manuscript with Track Changes'.

* An unmarked version of your revised paper without tracked changes. You should upload this as a separate file labeled 'Manuscript'.

If you would like to make changes to your financial disclosure, competing interests statement, or data availability statement, please make these updates within the submission form at the time of resubmission. Guidelines for resubmitting your figure files are available below the reviewer comments at the end of this letter

We look forward to receiving your revised manuscript.

Kind regards,

Tim Christian Kietzmann, Dr. rer. nat.

Academic Editor

PLOS Computational Biology

Marieke van Vugt

Section Editor

PLOS Computational Biology

**Journal Requirements:**

If the reviewer comments include a recommendation to cite specific previously published works, please review and evaluate these publications to determine whether they are relevant and should be cited. There is no requirement to cite these works unless the editor has indicated otherwise.

1) Please ensure that the CRediT author contributions listed for every co-author are completed accurately and in full.

At this stage, the following Authors/Authors require contributions: Charles Micou, and Timothy O'Leary. Please ensure that the full contributions of each author are acknowledged in the "Add/Edit/Remove Authors" section of our submission form.

The list of CRediT author contributions may be found here: https://journals.plos.org/ploscompbiol/s/authorship#loc-author-contributions

2) We ask that a manuscript source file is provided at Revision. Please upload your manuscript file as a .doc, .docx, .rtf or .tex. If you are providing a .tex file, please upload it under the item type u2018LaTeX Source Fileu2019 and leave your .pdf version as the item type u2018Manuscriptu2019.

3) Please provide an Author Summary. This should appear in your manuscript between the Abstract (if applicable) and the Introduction, and should be 150-200 words long. The aim should be to make your findings accessible to a wide audience that includes both scientists and non-scientists. Sample summaries can be found on our website under Submission Guidelines:

https://journals.plos.org/ploscompbiol/s/submission-guidelines#loc-parts-of-a-submission

4) Please upload all main figures as separate Figure files in .tif or .eps format. For more information about how to convert and format your figure files please see our guidelines:

https://journals.plos.org/ploscompbiol/s/figures

5) We notice that your supplementary Figures are included in the manuscript file. Please remove them and upload them with the file type 'Supporting Information'. Please ensure that each Supporting Information file has a legend listed in the manuscript after the references list.

6) Some material included in your submission may be copyrighted. According to PLOSu2019s copyright policy, authors who use figures or other material (e.g., graphics, clipart, maps) from another author or copyright holder must demonstrate or obtain permission to publish this material under the Creative Commons Attribution 4.0 International (CC BY 4.0) License used by PLOS journals. Please closely review the details of PLOSu2019s copyright requirements here: PLOS Licenses and Copyright. If you need to request permissions from a copyright holder, you may use PLOS's Copyright Content Permission form.

Please respond directly to this email and provide any known details concerning your material's license terms and permissions required for reuse, even if you have not yet obtained copyright permissions or are unsure of your material's copyright compatibility. Once you have responded and addressed all other outstanding technical requirements, you may resubmit your manuscript within Editorial Manager.

Potential Copyright Issues:

i) Figure 1. Please confirm whether you drew the images / clip-art within the figure panels by hand. If you did not draw the images, please provide (a) a link to the source of the images or icons and their license / terms of use; or (b) written permission from the copyright holder to publish the images or icons under our CC BY 4.0 license. Alternatively, you may replace the images with open source alternatives. See these open source resources you may use to replace images / clip-art:

- https://commons.wikimedia.org

- https://openclipart.org/.

7) Please provide a detailed Financial Disclosure statement. This is published with the article. It must therefore be completed in full sentences and contain the exact wording you wish to be published.

1) Please clarify all sources of financial support for your study. List the grants, grant numbers, and organizations that funded your study, including funding received from your institution. Please note that suppliers of material support, including research materials, should be recognized in the Acknowledgements section rather than in the Financial Disclosure

2) State the initials, alongside each funding source, of each author to receive each grant. For example: "This work was supported by the National Institutes of Health (####### to AM; ###### to CJ) and the National Science Foundation (###### to AM)."

3) State what role the funders took in the study. If the funders had no role in your study, please state: "The funders had no role in study design, data collection and analysis, decision to publish, or preparation of the manuscript."

4) If any authors received a salary from any of your funders, please state which authors and which funders..

If you did not receive any funding for this study, please simply state: u201cThe authors received no specific funding for this work.u201d

8) Please send a completed 'Competing Interests' statement, including any COIs declared by your co-authors. If you have no competing interests to declare, please state "The authors have declared that no competing interests exist". Otherwise please declare all competing interests beginning with the statement "I have read the journal's policy and the authors of this manuscript have the following competing interests"

**Reviewers' comments:**

Reviewer's Responses to Questions

**Comments to the Authors:**

**Please note here if the review is uploaded as an attachment.**

Reviewer #1: In this work, the authors begin by discussing representational drift and the idea that adaptive updating of downstream decoders can enable reliable readout despite gradually changing neural codes. They consider two single-cell-level models of drift: gradual drift in the tuning of the entire population, and abrupt, unbounded shifts in random subsets of neurons. Both models give rise to gradual drift at the population level, and their parameterizations allow comparison while controlling for the overall drift rate. The model involving abrupt tuning changes is shown to support significantly better unsupervised decoder adaptation than the gradual model. The authors further analyze two experimental datasets: recordings from primary visual cortex (V1) and posterior parietal cortex (PPC), both collected in studies designed to examine representational drift. By fitting a model combining gradual and abrupt changes to each dataset, they show that the abrupt component is more dominant in PPC than in V1.

Overall, I find this work interesting and timely. The last author has played a key role in conceptualizing and theoretically developing ideas surrounding representational drift and mechanisms for maintaining functionality despite it. The paper is solid, and the integration of theoretical modeling and data analysis to support a coherent hypothesis is much appreciated. While the models are intentionally simplified, as the authors explicitly note, I do not view this as a weakness, since it enables a sharp discussion of the central question. While abrupt single-cell switching in a given brain region has been documented previously, a key and novel contribution of this manuscript is the explicit formulation of representational drift as a weighted combination of gradual and abrupt components, and the use of that mixture model to compare drift regimes across brain areas. This framing goes beyond demonstrating the applicability of unsupervised decoder adaptation, and provides additional lens for asking whether and how different circuits employ fundamentally different modes of representational drift. Several issues require clearer explanation and additional analysis, and some notations would benefit from more precise definition.

Major comments:

1. The authors focus on analyzing pairwise correlations across neurons and the changes in these correlations between different days. What is the rationale for doing so, rather than examining correlations of the same cells across days (instead of the population of cell-pairs that were above a correlation threshold on the second day of comparison)?

Furthermore, the choice of using scaled correlation while including only cell-pairs whose correlation is above threshold on the later day is not explicitly motivated. This makes the choice appear somewhat arbitrary or even heavily tailored.

Including an illustration of the (2D) distribution of correlations in D1 and D2, and showing how thresholding and scaling yield a bimodal distribution, would help readers appreciate the rationale behind the definition of Delta C used in the paper.

2. While the bimodality in the distribution of the scaled correlation change is clear in both the simulation and the data, it still needs to be explicitly explained. Readers unfamiliar with this specific kind of analysis may find the reasoning behind the bimodality less clear. It would also be worth mentioning the relationship between bimodality and tuning-curve width (e.g., bimodality would likely be less prominent if tuning were cosine or thresholded cosine, rather than thresholded squared cosine as used here).

3. The authors have made a much-appreciated effort to include illustrative figures (Fig. 1, 2, 3a–b, etc.). It would be beneficial to have an additional schematic illustrating of the key conceptual point: for two systems in which the same overall level of drift is realized either through sparse abrupt changes or through global gradual changes, online correction by a downstream reader will be easier in the former.

4. A notable contribution of this work is its approach to parameterizing aspects of representational drift not captured by standard measures. Specifically, two systems may show the same overall rate of drift, yet one may drift gradually and the other abruptly. This distinction provides a framework for comparing drift across brain in a more nuanced way. It would be valuable to explicitly discuss this conceptual strength.

In the analysis, presenting the likelihood of different combinations of gradual and abrupt drift levels (given the data) could illustrate how accurately the contribution of each component can be estimated.

5. Using a Gaussian function may not be optimal for circular variables. The authors might consider using a von Mises distribution instead, which would offer a more natural and appropriate representation for this type of data.

6. The sensitivity of the reader’s adaptation to assumptions about drift parameters and stimulus priors should be further discussed and analyzed.

Minor comments:

1. In some places, the definitions should be refined for accuracy. For example:

The text refers to “correlation,” but the equation computes an inner product.

T_d is used in eq. 18 to define drift rate, while eq. 20 states “the population is scrambled on day T_d”; for consistency, clarify that T_d is the earliest day on which the population is scrambled.

x_i and x_j are sometimes referred to as neurons (page 6), though they are defined as rates.

In eq. 12, it is unclear whether the probability refers to x_i,x_j themselves or to the similarity between them.

It is unclear whether sigma in eq. 9 and eq. 14 denotes the same parameter.

Given independent noise across neurons (eq. 1), the notation should likely be xi_i, not just xi.

2. The introduction says “and how these limitations are mitigated by heavy-tailed statistics”, at this early point of the paper, it may be worth explicitly stating that the statement refer to.

2. Provide specific examples of single cells from the datasets illustrating gradual vs. abrupt drift, even if only for illustrative purposes.

3. The manuscript states that “representational drift generically leads to a gradual degradation in the accuracy of a fixed readout… eventually rendering that readout no better than chance.” This is true for some reports but not all; some studies have shown preserved decoding despite drift. (Still, adaptive readout updates are important either way.)

4. The phrase “Finally, we explore whether biology makes use of these advantageous statistics…” may suggest a mechanistic claim (that biology uses the statistics), rather than an observational one. Consider replacing “makes use of” with “exhibits” or “manifests.”

5. When PPC is first mentioned, briefly describe it or its functions (Unlike “visual cortex,” its role is not self-explanatory).

6. Figure 1a is clear, but Figure 1b is not. It seems to conflate two ideas, the reader’s update and the ambiguity between noise and representational drift.

7. The phrase “nonstationary statistics” is technically correct (as distinct from noise) but may be misleading, since drift may still have stationary statistics in some senses (unlike learning).

8. In Fig. 1b., the horizontal dashed lines’ meaning is unclear and they resemble the neuron frames too closely.

9. For the notation for smallest angle between psi_1 and psi_2, defining it in an equation instead of an inline definition, would be clearer.

10. For eq. 11, include a reference to the supplementary derivations.

11. “knowledge of how the population was tuned on an earlier day, as well as a drift prior describing how that tuning is likely to change, is required to anchor the system to an absolute frame of reference.” I would guess that the assumption of drift being generally slow may be as important as specific priors.

12. In some figures (e.g., 2a, 3a), day 1 uses the ground truth, while in others (e.g., 3d, 3e) there appear to be errors already on day 1 (so initialization is at day 0). Clarify for consistency.

13. The phrase “the two models of drift provide identical long-term behaviour”: does it mean convergence to the same steady state, or identical functions of time for large t? Clarify the intended meaning.

14. After eq. 21, explicitly explain the notations C_D1 and C_D2.

15. The sentence “changes in the distribution of tuning do not fit neatly into the mould of either sudden or gradual drift (Fig. 4f)” could be revised to: “changes in the distribution of tuning do not fit neatly into the model of either sudden (Fig. 4e) or gradual drift (Fig. 4f)”.

16. Change some phrases to be clearer: “and to be have well-correlated activity” to “and to have well-correlated activity”; “do not have direct access external world” to “do not have direct access to the external world”; and “To explore whether in-drift manifests drift suddenly or gradually” to ”To explore whether (in-vivo?) drift is manifested suddenly or gradually”.

17. The statement “The population with the slower drift rate makes a greater proportion of tuning changes gradually”: clarify what was statistically tested, and consider softening the claim given the limited significance (as it wasn’t significant in one analysis and p values was 0.034 in another).

18. Maybe change the phrase “an argument and computational simulations” to “an analytical argument and simulations” (as any simulation is computational).

19. In the phrase “this advantage is more pronounced in situations involving higher drift rates”, would it be correct to mention both higher drift rates and smaller population (as also discussed in Fig. 3g)?

20. The important point that an observation of gradual drift at population-level (and after averaging across-animal and across day-pairs averaging) does not necessarily imply gradual drift at the single-cell level appears only in the final paragraph. It deserves mention earlier in the paper, possibly in the Introduction.

Reviewer #2: Representational drift is a common observation across many brain regions. If neurons continually change their tuning properties, how can downstream circuits correctly interpret their activity? Micou and O’Leary address this question by proposing that the brain can exploit stimulus-evoked correlations between neurons to continually infer shifting tuning properties in an unsupervised manner. Using a simple model, they compare two forms of representational drift: (i) gradual, approximately homogeneous drift across the population, akin to Brownian motion; and (ii) abrupt, large changes restricted to a small subset of neurons. They show that their proposed mechanism performs better under the latter form of drift. Finally, using neural data from two brain regions, they argue that the experimentally observed drift is more consistent with the sparse, abrupt type.

Overall, I found the paper original and interesting. My comments are primarily minor, but addressing them can improve clarity and precision.

Specific Comments

Page 3:

The sentence beginning “Drift is distinguished from the other form of nonstationary changes…” is very long and difficult to parse. Its meaning is unclear to me. I recommend breaking it into multiple shorter sentences.

Pages 3–4: Notation

The use of boldface notation for vectors is inconsistent. For example, (\mathbf{s}) and (\mathbf{x}) are vectors and are typeset in bold, but (\theta_i), which appears to be a vector in (\mathbb{R}^P), is not bolded in Equation (1). If (\theta_i) is intended to be a vector, it should be written as (\boldsymbol{\theta}_i) for consistency, unless there is a conceptual reason for the distinction that should be clarified.

Page 6:

The gradual drift is described as an Ornstein–Uhlenbeck (OU) process. However, OU dynamics revert to a mean, whereas Brownian motion on the circle does not. If the intended process lacks mean reversion, then describing it as OU is inaccurate. Please clarify the stochastic dynamics underlying the “gradual” drift condition.

Equations (16) and (17):

The quantities (f_{\mathrm{gradual}}) and (f_{\mathrm{sudden}}) appear in the equations but are not introduced in the text. A brief explanation of what these functions represent would help readers follow the development of the model.

Equation (17):

The “0 otherwise” clause seems unnecessary, since the preceding line already specifies the value for all (\theta_{i,t}).

Equation (20):

The definition of T_d with inequality is inaccurate. Either use equality, or define T_d as the minimal number of days such that the inequality is satisfied.

Discussion

I found the arguments in the first paragraph of the Discussion section and in the in-silico section of the Discussion sections confusing. There is no evidence that representational drift is a feature and not a bug of the system. The authors have shown that abrupt changes in few neurons are less detrimental than gradual ones in all neurons. However, to argue for the “advantages of …changes in representation”, and their possible role in silco, one first needs to show convincing evidence that these changes are advantageous in the first place.

Additional Suggestion

An intuitive explanation might help readers before introducing the formalism. Suppose the neural population is large and noise levels are low. At each time step, only a small fraction of neurons undergo substantial tuning changes. Because these changes induce noticeable shifts in their correlations with other neurons, these neurons are easy to identify. Their new tuning curves could then be inferred by examining the tuning of the neurons with which they are now correlated. This intuition may provide a helpful conceptual bridge to the correlation-based inference approach used in the paper.

Yonatan Loewenstein

Reviewer #3: In the manuscript, “Statistics of cortical representational drift can enable robust readout,” the authors propose an adaptive decoding scheme which allows one to overcome the degradation in readout due to representational drift (RD). The scheme is meant as an ideal normative solution to drifting population codes, and not as a mechanistic description of a physiological mechanism for readout. The main finding is that, given a fixed amount of drift over a certain period of time, the decoder works better if some neurons undergo significant drift and others much less, compared to the scenario where all cells undergo a bit of drift. Analysis of data from parietal and visual cortices reveal a mixed bag, where neither the “all-a-little-bit” Gaussian model, nor the broad-tailed “stay-or-change-a-lot” model capture the statistics of drift alone (as characterized by changes in pairwise correlations). Rather, a mixture of the two does well, with broad-tailed changes in tuning doing better when drift is fast.

What a pleasure it is to read a well-written paper, with clearly expressed ideas and an interesting and not-overstated main finding. This is an important contribution to the literature on RD and I have relatively little to say beyond that.

I do, however, have some thoughts on the relationship between the mechanisms responsible for drift, and the changes in the parameters “theta” in this manuscript. This is something the authors very clearly acknowledge is -not- the topic of the current manuscript, however I believe it is germane to the conclusions drawn. Namely, at least for the data sets analyzed, the authors can rule out simple Gaussian statistics for the RD, particularly for the parietal cortex. However, the observed changes in cells’ tuning are likely due to either synaptic plasticity or single-cell excitability, both of which will affect the cells’ tuning in nonlinear ways. Put another way, assuming changes in a cell’s input follow Gaussian statistics, it is quite possible that the consequent changes in tuning are -not- Gaussian. At least in the case of a cell’s firing rate, it is known that a Gaussian distribution of inputs actually results in a log-normal, i.e. long-tailed, distribution of firing rates. Recent work with spiking networks (Devalle et al, Sci Rep 2025) showed that Gaussian changes in inputs are sufficient to provide a quantitative fit to data from hippocampus, though perhaps importantly pairwise statistics were not studied. So there is some chance that simple Gaussian statistics at the mechanistic level of description, combined with whatever nonlinearities transform this into changes in the “thetas” could account for RD in the data sets looked at here.

That’s it. I’m very happy to hear the authors’ thoughts on this, whether or not they feel it warrants any change to the main text.

Minor typo: Fourth line from the end on page two, “used changed in” should be “used changes in” I believe.

**********

**Have the authors made all data and (if applicable) computational code underlying the findings in their manuscript fully available?**

The PLOS Data policy requires authors to make all data and code underlying the findings described in their manuscript fully available without restriction, with rare exception (please refer to the Data Availability Statement in the manuscript PDF file). The data and code should be provided as part of the manuscript or its supporting information, or deposited to a public repository. For example, in addition to summary statistics, the data points behind means, medians and variance measures should be available. If there are restrictions on publicly sharing data or code —e.g. participant privacy or use of data from a third party—those must be specified.

Reviewer #1: Yes

Reviewer #2: None

Reviewer #3: Yes

**********

PLOS authors have the option to publish the peer review history of their article (what does this mean?). If published, this will include your full peer review and any attached files.

If you choose “no”, your identity will remain anonymous but your review may still be made public.

**Do you want your identity to be public for this peer review?**    For information about this choice, including consent withdrawal, please see our Privacy Policy.

Reviewer #1: No

Reviewer #2: No

Reviewer #3: No

**Figure resubmission:**

While revising your submission, we strongly recommend that you use PLOS’s NAAS tool (https://ngplosjournals.pagemajik.ai/artanalysis) to test your figure files. NAAS can convert your figure files to the TIFF file type and meet basic requirements (such as print size, resolution), or provide you with a report on issues that do not meet our requirements and that NAAS cannot fix.-->

After uploading your figures to PLOS’s NAAS tool - https://ngplosjournals.pagemajik.ai/artanalysis, NAAS will process the files provided and display the results in the "Uploaded Files" section of the page as the processing is complete. If the uploaded figures meet our requirements (or NAAS is able to fix the files to meet our requirements), the figure will be marked as "fixed" above. If NAAS is unable to fix the files, a red "failed" label will appear above. When NAAS has confirmed that the figure files meet our requirements, please download the file via the download option, and include these NAAS processed figure files when submitting your revised manuscript.

**Reproducibility:**

To enhance the reproducibility of your results, we recommend that authors of applicable studies deposit laboratory protocols in protocols.io, where a protocol can be assigned its own identifier (DOI) such that it can be cited independently in the future. Additionally, PLOS ONE offers an option to publish peer-reviewed clinical study protocols. Read more information on sharing protocols at https://plos.org/protocols?utm_medium=editorial-email&utm_source=authorletters&utm_campaign=protocols

# Author comment: Author response to Decision Letter 1

**DOI:** 10.1371/journal.pcbi.1014297.r002

27 Mar 2026

Attachment Submitted filename: Responses to reviewers.docx

# Peer review: Decision Letter 1

**DOI:** 10.1371/journal.pcbi.1014297.r003

**Authors:** Tim Kietzmann

5 May 2026

Dear Prof O'Leary,

We are pleased to inform you that your manuscript 'Statistics of cortical representational drift can enable robust readout' has been provisionally accepted for publication in PLOS Computational Biology.

Before your manuscript can be formally accepted you will need to complete some formatting changes, which you will receive in a follow up email. A member of our team will be in touch with a set of requests.

Please note that your manuscript will not be scheduled for publication until you have made the required changes, so a swift response is appreciated.

IMPORTANT: The editorial review process is now complete. PLOS will only permit corrections to spelling, formatting or significant scientific errors from this point onwards. Requests for major changes, or any which affect the scientific understanding of your work, will cause delays to the publication date of your manuscript.

Should you, your institution's press office or the journal office choose to press release your paper, you will automatically be opted out of early publication. We ask that you notify us now if you or your institution is planning to press release the article. All press must be co-ordinated with PLOS.

Thank you again for supporting Open Access publishing; we are looking forward to publishing your work in PLOS Computational Biology.

Best regards,

Tim Christian Kietzmann, Dr. rer. nat.

Academic Editor

PLOS Computational Biology

Marieke van Vugt

Section Editor

PLOS Computational Biology

***********************************************************

Reviewer's Responses to Questions

**Comments to the Authors:**

**Please note here if the review is uploaded as an attachment.**

Reviewer #1: I thank the authors for their thorough responses to my comments. This is a very nice piece of work, and I look forward to seeing it in press.

Please note that there appears to be a technical issue with the references.

Reviewer #2: The authors have carefully and successfully addressed all of my concerns, and I am pleased to recommend the revised manuscript for publication.

Reviewer #3: I am happy for the manuscript to be published as is.

**********

**Have the authors made all data and (if applicable) computational code underlying the findings in their manuscript fully available?**

The PLOS Data policy requires authors to make all data and code underlying the findings described in their manuscript fully available without restriction, with rare exception (please refer to the Data Availability Statement in the manuscript PDF file). The data and code should be provided as part of the manuscript or its supporting information, or deposited to a public repository. For example, in addition to summary statistics, the data points behind means, medians and variance measures should be available. If there are restrictions on publicly sharing data or code —e.g. participant privacy or use of data from a third party—those must be specified.

Reviewer #1: Yes

Reviewer #2: None

Reviewer #3: None

**********

PLOS authors have the option to publish the peer review history of their article (what does this mean?). If published, this will include your full peer review and any attached files.

If you choose “no”, your identity will remain anonymous but your review may still be made public.

**Do you want your identity to be public for this peer review?**    For information about this choice, including consent withdrawal, please see our Privacy Policy.

Reviewer #1: No

Reviewer #2: **Yes:**   Yonatan Loewenstein

Reviewer #3: No

# Editor report: Acceptance letter

**DOI:** 10.1371/journal.pcbi.1014297.r004

**Authors:** Tim Kietzmann

PCOMPBIOL-D-25-01957R1

Statistics of cortical representational drift can enable robust readout

Dear Dr O'Leary,

I am pleased to inform you that your manuscript has been formally accepted for publication in PLOS Computational Biology. Your manuscript is now with our production department and you will be notified of the publication date in due course.

The corresponding author will soon be receiving a typeset proof for review, to ensure errors have not been introduced during production. Please review the PDF proof of your manuscript carefully, as this is the last chance to correct any errors. Please note that major changes, or those which affect the scientific understanding of the work, will likely cause delays to the publication date of your manuscript.

Soon after your final files are uploaded, unless you have opted out, the early version of your manuscript will be published online. The date of the early version will be your article's publication date. The final article will be published to the same URL, and all versions of the paper will be accessible to readers.

For Research, Software, and Methods articles, you will receive an invoice from PLOS for your publication fee after your manuscript has reached the completed accept phase. If you receive an email requesting payment before acceptance or for any other service, this may be a phishing scheme. Learn how to identify phishing emails and protect your accounts at https://explore.plos.org/phishing.

Thank you again for supporting PLOS Computational Biology and open-access publishing. We are looking forward to publishing your work!

With kind regards,

Anita Estes

PLOS Computational Biology | Carlyle House, Carlyle Road, Cambridge CB4 3DN | United Kingdom ploscompbiol@plos.org | Phone +44 (0) 1223-442824 | ploscompbiol.org | @PLOSCompBiol
